# Role of Ubiquitin-regulated EMT in Cancer Metastasis and Chemoresistance

**DOI:** 10.7150/ijbs.115401

**Published:** 2025-09-29

**Authors:** Shuai Xiao, Lingli Tian, Xiaoli Gan, Xiaozhen Xu, Meng Liao, Dandan Song, Yeping Yu, Wenying Qin, Rui Zhang, Hao Lyu, Dong Guo, Qi Zhang, Xing-Zhen Chen, Cefan Zhou, Jingfeng Tang

**Affiliations:** 1 National “111” Center for Cellular Regulation and Molecular Pharmaceutics, Hubei University of Technology, Wuhan 430068, China.; 2 Hubei Key Laboratory of Industrial Microbiology, Hubei University of Technology, Wuhan 430068, China.; 3 School of Life Sciences, Zhengzhou University, Zhengzhou 450001, China.; 4 Membrane Protein Disease Research Group, Department of Physiology, Faculty of Medicine and Dentistry, University of Alberta, Edmonton, AB T6G 2R3, Canada.

**Keywords:** Ubiquitination, Epithelial-mesenchymal transition, tumor metastasis, Drug resistance

## Abstract

Epithelial-mesenchymal transition (EMT) is a fundamental biological process that promotes cancer metastasis and chemoresistance. However, the therapeutic efficacy of EMT inhibitors remains limited. Ubiquitination, a critical post-translational modification, involves attaching ubiquitin molecules to proteins to regulate their function and stability. It modulates EMT by controlling key EMT transcription factors (EMT-TFs) and associated signaling pathways. Evidence indicates that ubiquitination-dependent regulation of EMT serves as a central mechanism underlying tumor metastasis and chemoresistance. Targeting specific deubiquitinases (DUBs) or E3 ligases can effectively reverse EMT-induced cancer progression and treatment resistance. These findings highlight the therapeutic potential of E3 ligase and DUB inhibitors in oncology. Collectively, ubiquitination-regulated EMT is pivotal in mediating metastasis and chemoresistance in malignant tumors. This review summarizes the molecular mechanisms of EMT and emphasizes ubiquitination's essential role in regulating EMT to promote tumor metastasis and chemoresistance. Consequently, developing inhibitors against specific E3 ligases and DUBs offers a promising strategy to improve cancer treatment outcomes.

## Introduction

**Figure 1:**
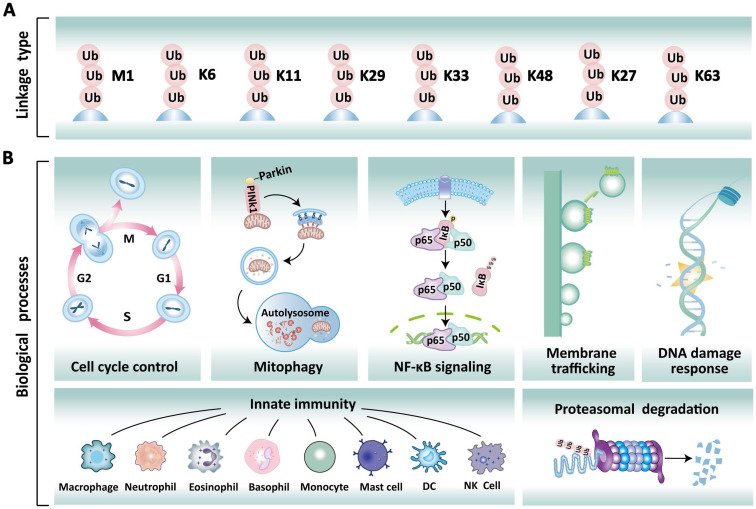
** Different types of ubiquitinated chains and various physiological roles. (A)** Ubiquitin chain can be classified into eight based on linkage types: Met1, K6, K11, K27, K29, K33, K48, and K63. **(B)** Different ubiquitination modifications play a specific role in various cellular processes, including cell cycle regulation, mitophagy, NF-κB signaling, membrane trafficking, DNA damage repair, innate immune response, and proteasomal degradation.

Ubiquitination is a fundamental and reversible post-translational modification (PTM) that plays a pivotal role in eukaryotic cellular homeostasis by dynamically regulating protein stability, activity, localization, and function 1. This modification process primarily involves ubiquitin molecules, which consist of 76 amino acids and are highly conserved across eukaryotes 2. In mammalian cells, polyubiquitination typically occurs through the conjugation of ubiquitin via the initial methionine (M1) and seven lysine residues (K6, K11, K27, K29, K33, K48, K63) (Figure 1A) 3. These distinct ubiquitin modifications exhibit functional diversity. For instance, K48- and K11-linked polyubiquitination predominantly serve as proteolytic signals directing 26S proteasome-mediated substrate recognition and degradation 4, 5. In contrast, K63-linked polyubiquitination can influence the functions of proteins involved in DNA damage response, signal transduction, and cell cycle control 6. Notably, the M1-linked linear ubiquitination is formed through the N-terminal methionine residue of ubiquitin, playing a pivotal role in immune regulation and inflammatory responses by activating the NF-κB transcription factor 5. Furthermore, the remaining linkage types (K6, K27, K29, K33) exhibit pleiotropic roles that span protein activity modulation, intracellular signaling, genomic stability maintenance, cell cycle checkpoint control, and innate immune regulation 6, 7. Deubiquitination is the reverse reaction catalyzed by deubiquitinases (DUBs), which remove ubiquitin chains to stabilize proteins and modulate biological processes. Collectively, the dynamic balance between ubiquitination and deubiquitination is crucial for maintaining the normal physiological functions of the cell, including protein degradation, DNA damage repair, the cell cycle, and signal transduction (Figure 1B).

**Figure 2:**
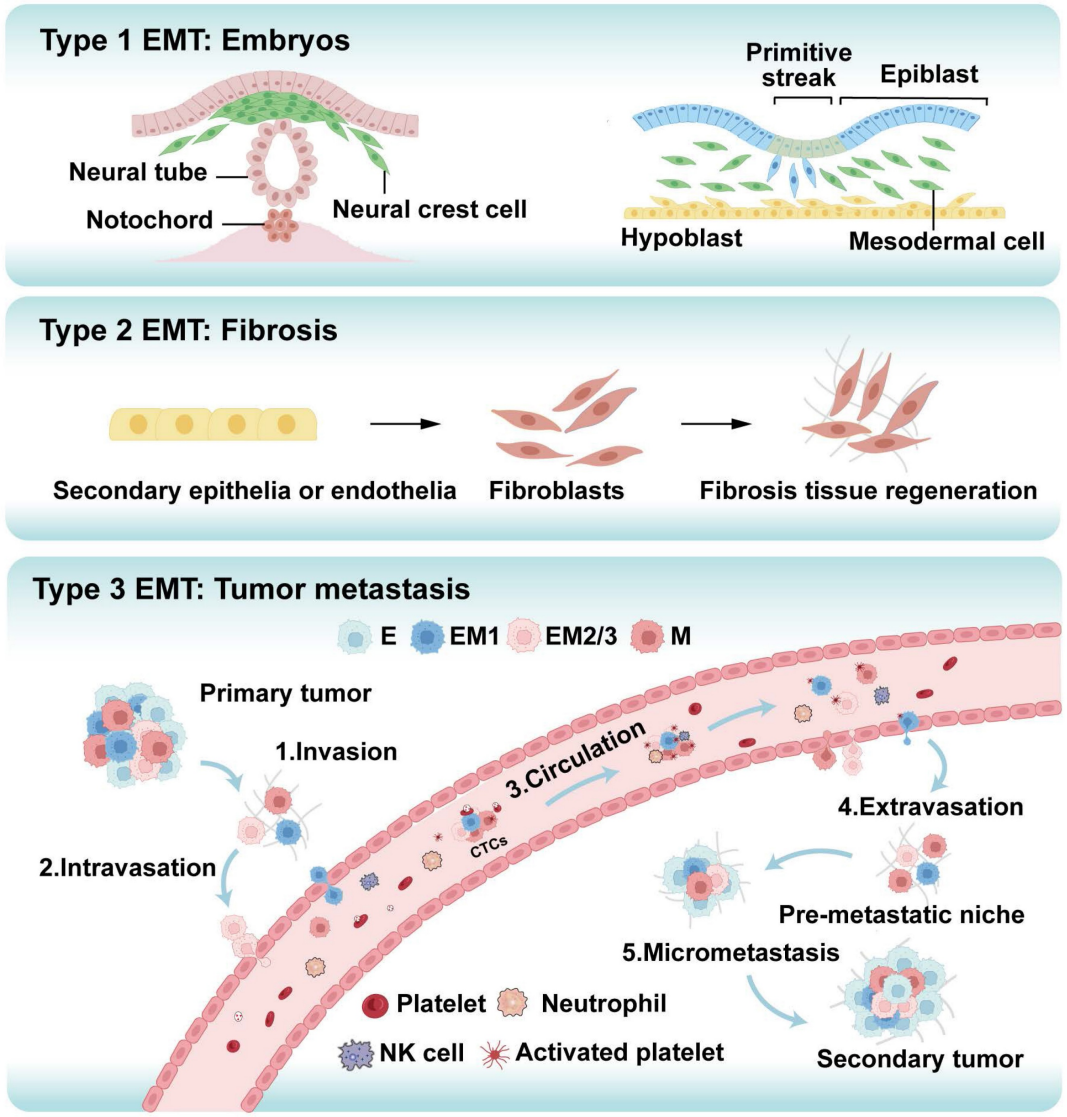
** The three types of epithelial-mesenchymal transition (EMT) play distinct yet significant roles in various biological processes.** Type 1 EMT is crucial for embryonic development, facilitating cell migration and differentiation, which are essential for processes such as gastrulation, neural crest cell migration, and organogenesis. Type 2 EMT is associated with fibrosis, promoting the transformation of epithelial cells into mesenchymal cells, which leads to excessive extracellular matrix (ECM) deposition, tissue remodeling, and ultimately organ dysfunction. Type 3 EMT is particularly critical in cancer metastasis, where primary tumor cells acquire invasive capabilities through EMT. These cells detach from the primary site and undergo five key steps: local invasion, intravasation, survival in the circulation, extravasation, and the formation of metastatic lesions. This process enables the spread of cancer to other parts of the body, resulting in the establishment of secondary tumors or metastatic disease. E: epithelial tumor cell, M: mesenchymal tumor cell, EM1, EM2/3:intermediate cell states, CTCs: circulating tumor cells.

Epithelial-Mesenchymal Transition (EMT) is a cellular reprogramming process in which epithelial cells lose their polarity and cell-cell adhesion properties while acquiring migratory and invasive mesenchymal characteristics 8. This process, initially characterized in embryonic development and wound healing, is hijacked during cancer progression to drive metastasis, the leading cause of cancer-related mortality 9. EMT is classified into three distinct types: Type 1 EMT governs normal developmental processes such as embryogenesis; Type 2 EMT facilitates tissue repair and is linked to inflammation and fibrosis; Type 3 EMT, associated with tumor progression, promotes invasion and metastasis (Figure 2) 10-12. In oncology, EMT enables tumor cells to disseminate from the primary site, enhance invasiveness, and initiate systemic spread 13-15. This process enhances cellular plasticity, allowing for transitions to hybrid epithelial/mesenchymal states that are highly aggressive and prone to metastasis 16. Cytoskeletal reorganization during EMT alters cell shape and motility, promoting migration. Moreover, EMT activates cancer stem cell (CSC) properties, upregulating stemness genes that amplify metastatic potential and confer treatment resistance, partly due to acquired mesenchymal resilience to therapies 17, 18. Overall, EMT promotes metastasis by enabling profound cellular plasticity.

Ubiquitination critically regulates EMT by controlling the degradation and stability of key proteins through the ubiquitin-proteasome system (UPS) 19. This process is primarily mediated by E3 ligases and DUBs, which regulate the key EMT transcription factors (EMT-TFs) and EMT-associated signaling pathways 19. Notably, the stability of Snail, a critical EMT transcription factor, is dynamically controlled by ubiquitination; dysregulation of this process enhances EMT and cancer progression 20. For instance, in colorectal cancer (CRC), mitogen and stress-activated protein kinase 1 (MSK1) recruits USP5 to deubiquitinate and stabilize Snail, facilitating EMT and metastasis 21. Conversely, in triple-negative breast cancer (TNBC), the E3 ligase Membrane Associated Ring-CH-Type Finger 2 (MARCH2) ubiquitinates Snail, driving its degradation and suppressing tumor growth and metastasis 22. In non-small cell lung cancer (NSCLC), RNF187 promotes EMT and apoptosis resistance by activating MAPK/PI3K signaling pathways 23. Dysregulated expression of ubiquitination-related enzymes, such as E3 ligases or DUBs, frequently promotes EMT activation across various cancers, driving enhanced cell migration, invasion, metastasis, and therapy resistance 24, 25. Therefore, ubiquitination profoundly influences tumor invasion, metastasis, and drug resistance by modulating EMT dynamics. This review summarizes the molecular mechanisms of EMT and highlights its pivotal roles in driving tumor metastasis and chemoresistance. We further elaborate on how EMT is regulated by specific E3 ligases and DUBs, which modulate key EMT inducers like ZEB1 and Snail via ubiquitination-mediated degradation or stabilization. Notably, targeting specific E3 ligases or DUBs can reverse EMT-associated metastasis and chemoresistance. These insights enhance our understanding of ubiquitination in EMT-driven tumor metastasis and drug resistance, supporting the development of E3 ligase or DUB inhibitors as promising antitumor therapies.

## Overview of ubiquitination and deubiquitination

**Figure 3:**
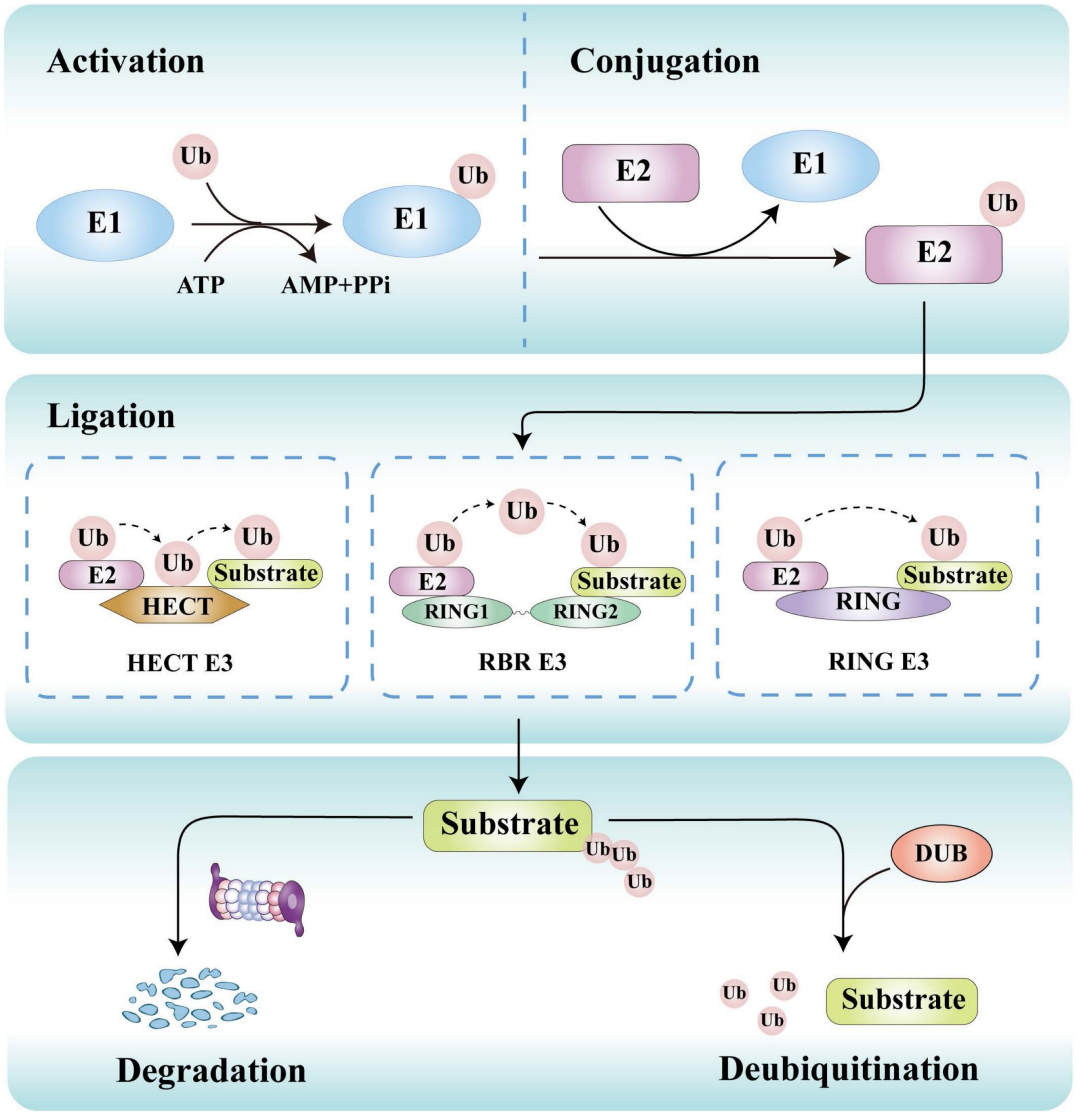
** Ubiquitination is a dynamic and reversible process.** The process of ubiquitin transfer necessitates the coordinated action of three types of ubiquitinating enzymes. Initially, the E1 ubiquitin-activating enzyme activates ubiquitin in an ATP-dependent manner. Subsequently, the activated ubiquitin molecule is transferred from the E1 enzyme to E2 ubiquitin-conjugating enzymes. Finally, E3 ubiquitin ligases facilitate the transfer of ubiquitin from E2 to target substrates, which may occur either directly or indirectly, depending on their structural characteristics and functional roles. Conversely, deubiquitinases (DUBs) can remove ubiquitin from substrates, thereby regulating the level of ubiquitination and the stability of proteins.

Ubiquitination is initiated by E1 activating enzymes that form a thioester bond with ubiquitin in an ATP-dependent manner. This ubiquitin is then transferred to E2 conjugating enzymes, which collaborate with E3 ligases to attach ubiquitin to lysine residues or other sites on substrate proteins (Figure 3) 26. This process can lead to proteasomal degradation or functional alterations of target proteins. The primary function of E1 activating enzymes is to initiate the activation process of ubiquitin molecules. Currently, eight distinct E1 enzymes have been identified, including conventional enzymes such as UBA1, UBA6, UBA7, SAE, and NAE, as well as non-conventional enzymes like UBA4, UBA5, and ATG7 27. Around 40 E2 enzymes have been identified, which are responsible for selecting the specific lysine residues located on the target protein that will undergo covalent binding with the ubiquitin molecule 28. E3 ligases play a critical role in determining substrate specificity by directly recognizing the target protein 29. More than 800 E3 ligases have been discovered, which are classified into three distinct families: the really interesting new gene (RING), homologous to E6AP carboxyl terminus (HECT), and RING-between-RING (RBR) E3 ligases 30. They employ unique catalytic strategies to regulate protein fate.

Deubiquitination counterbalances ubiquitination through removing ubiquitin from substrate proteins by a family of over 100 DUBs, which catalyze the hydrolysis of ubiquitin chains from substrates 31-33. DUBs are implicated in a wide range of cellular processes, such as protein stability regulation, signal transduction, cell cycle control, DNA repair, and tumorigenesis 34. These enzymes can be categorized into seven primary families: ubiquitin-specific proteases (USPs), ubiquitin carboxy-terminal hydrolases (UCHs), motif interacting with ubiquitin-containing novel DUB (MINDYs), JAMM/MPN domain-associated metallopeptidases (JAMMs), ovarian tumor-related proteases (OTUs), Machado-Joseph domain proteases (MJDs), and Zinc finger and UFSP domain protein (ZUFSP) 35. Dysregulation of DUB activity is implicated in various diseases, particularly tumors 36 and neurodegenerative diseases 37. DUBs have been shown to influence tumor progression and metastasis, with some DUBs acting as inhibitors while others promote tumor development. For instance, USP10 stabilizes p53 by deubiquitinating, thus counteracting Murine double minute 2 (MDM2)-mediated ubiquitination and inhibiting the growth of renal cell carcinoma (RCC) cells 38. Furthermore, USP14 stabilizes the oncogene protein B-cell lymphoma 6 through deubiquitination, thereby promoting the proliferation of ovarian cancer (OC) cells 39. Collectively, dysregulation of ubiquitination or deubiquitination disrupts this homeostasis, contributing to pathological conditions such as cancer, neurodegenerative disorders, cardiovascular diseases, and metabolic syndromes.

## EMT: Mechanisms and roles in cancer

The EMT process involves key molecular alterations, including the downregulation of epithelial markers and the upregulation of mesenchymal markers, which collectively modify cell adhesion properties 40. These molecular shifts are intrinsically coupled with cytoskeletal remodeling, directly impacting cellular morphology and motility. The EMT is regulated by key EMT-TFs, signaling pathways, and epigenetic modifications that influence gene expression changes 41, 42. Among these EMT-TFs, zinc-finger proteins (Snail and Slug), zinc-finger E-box binding homeobox factors (ZEB1 and ZEB2), and basic helix-loop-helix proteins (Twist1 and Twist2) have been studied the most extensively 43, 44. Specifically, Snail and Slug bind to the E-box motif in the *CDH1* promoter region to repress E-cadherin expression while activating mesenchymal gene transcription, thereby driving EMT progression 45, 46. Similarly, Twist1 and Twist2 suppress epithelial genes and promote the expression of mesenchymal genes, contributing to EMT-associated metastasis 47. ZEB1 and ZEB2 further inhibit *CDH1* transcription via E-box binding, accelerating the transition to a mesenchymal phenotype 48, 49. Collectively, these TFs orchestrate the expression of mesenchymal phenotypic markers and underpin the molecular dynamics of EMT.

Furthermore, the regulation of EMT involves multiple signaling pathways, including the TGF-β, Wnt/β-catenin, Notch, Hedgehog (Hh), and Hypoxia signaling 50. These extracellular signals promote the transcription of EMT-TF, thereby regulating the EMT process 50. Specifically, TGF-β signaling induces morphological and functional alterations in cells through both SMAD-dependent and non-SMAD-dependent mechanisms 51. In the Smad-dependent pathway, TGF-β activation results in the transcriptional regulation of EMT-TFs, facilitating the downregulation of epithelial markers and upregulation of mesenchymal markers 52. Additionally, in non-SMAD pathways, TGF-β engages MAPK (including ERK, JNK, and p38), Rho-like GTPase, and PI3K/AKT signaling to modulate EMT 53-55. The Wnt/β-catenin signaling pathway plays a critical role in stabilizing EMT-TFs and enhancing the transcription of EMT-related genes, contributing to metastasis 56. Under hypoxic conditions, Notch signaling amplifies hypoxia-inducible factor-1α (HIF-1α)-mediated activation of the *lysyl oxidase (LOX)* gene, which facilitates Snail expression and EMT progression 57. Furthermore, Notch signaling interacts with Wnt and TGF-β signaling, jointly regulating the expression of EMT-TF and enhancing the EMT in tumor contexts 58. Hh signaling activation occurs via ligand binding, leading to Gli transcription factor nuclear translocation and subsequent regulation of EMT-TFs, including Snail and Twist family members 59, 60.

EMT involves the transformation of epithelial cells into mesenchymal phenotypes, enhancing cell motility, invasiveness, and stemness 61. This process is reactivated in cancers and directly contributes to tumor metastasis and treatment resistance 62, 63. Mechanistically, key signaling pathways (TGF-β, Wnt/β-catenin, Notch, Hh, and Hypoxia) promote the expression of EMT-TFs (Snail, Twist, ZEB), which collectively repress E-cadherin while upregulating mesenchymal markers like vimentin and N-cadherin 62, 64-66. Matrix metalloproteinases (MMPs) facilitate invasion by degrading extracellular matrix components and activating EMT-associated signals 67. EMT also enhances cellular adaptability within hypoxic tumor microenvironments, contributing to survival under metabolic stress 68. Beyond promoting motility and invasiveness, EMT is linked to increased stemness and the activation of anti-apoptotic mechanisms and multidrug resistance efflux pumps, which together heighten tumor heterogeneity and treatment resistance 69. Moreover, EMT cooperates with immunosuppressive elements in the tumor microenvironment, reducing sensitivity to immunotherapies. In NSCLC, EMT-induced immunosuppression correlates with poor patient outcomes 70. Furthermore, in mesenchymal tumors such as osteosarcoma (OS), the EMT phenotype is also associated with chemotherapy resistance 69, 71. Furthermore, the high expression of EMT-TFs such as Snail and Slug significantly enhanced the cisplatin resistance in ovarian carcinoma (OC) 72. Collectively, the EMT process is closely related to tumor cell invasion, metastasis, and treatment resistance.

## Ubiquitination in regulating EMT

**Figure 4:**
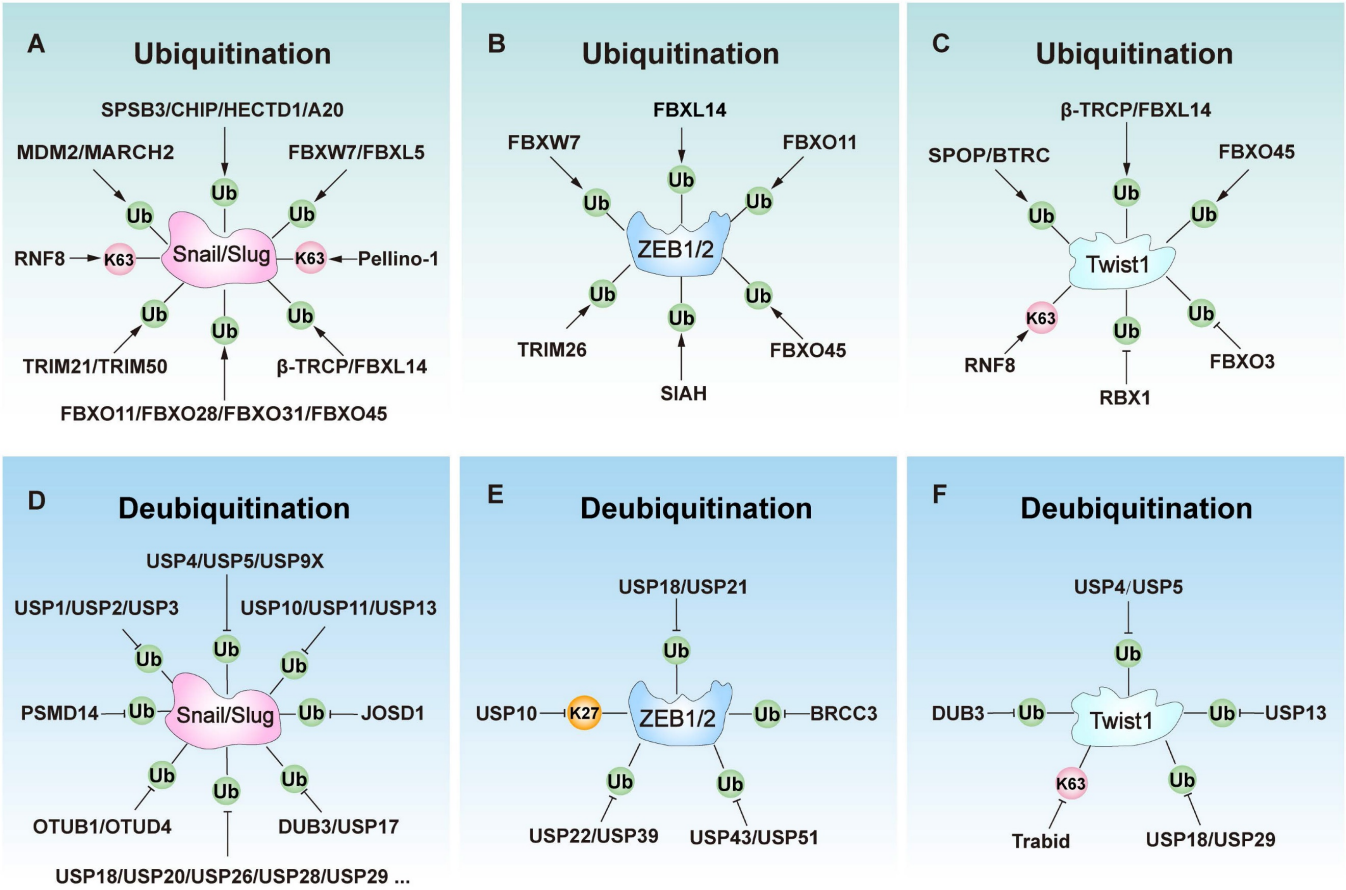
** The role of E3s and deubiquitinases (DUBs) involves key transcript factors in Epithelial-mesenchymal transition (EMT) regulation, including Snail, Slug, ZEB1, ZEB2, and Twist1. (A)** E3 ligases ubiquitinate Slug or Snail to regulate EMT. **(B)** E3 ligases ubiquitinate ZEB1 or ZEB2 to regulate EMT. **(C)** DUBs ubiquitinate Twist1 to regulate EMT. **(D)** DUBs ubiquitinate Slug or Snail to regulate EMT. **(E)** E3 ligases ubiquitinate ZEB1 or ZEB2 to regulate EMT. **(F)** DUBs ubiquitinate Twist1 to regulate EMT.

**Figure 5:**
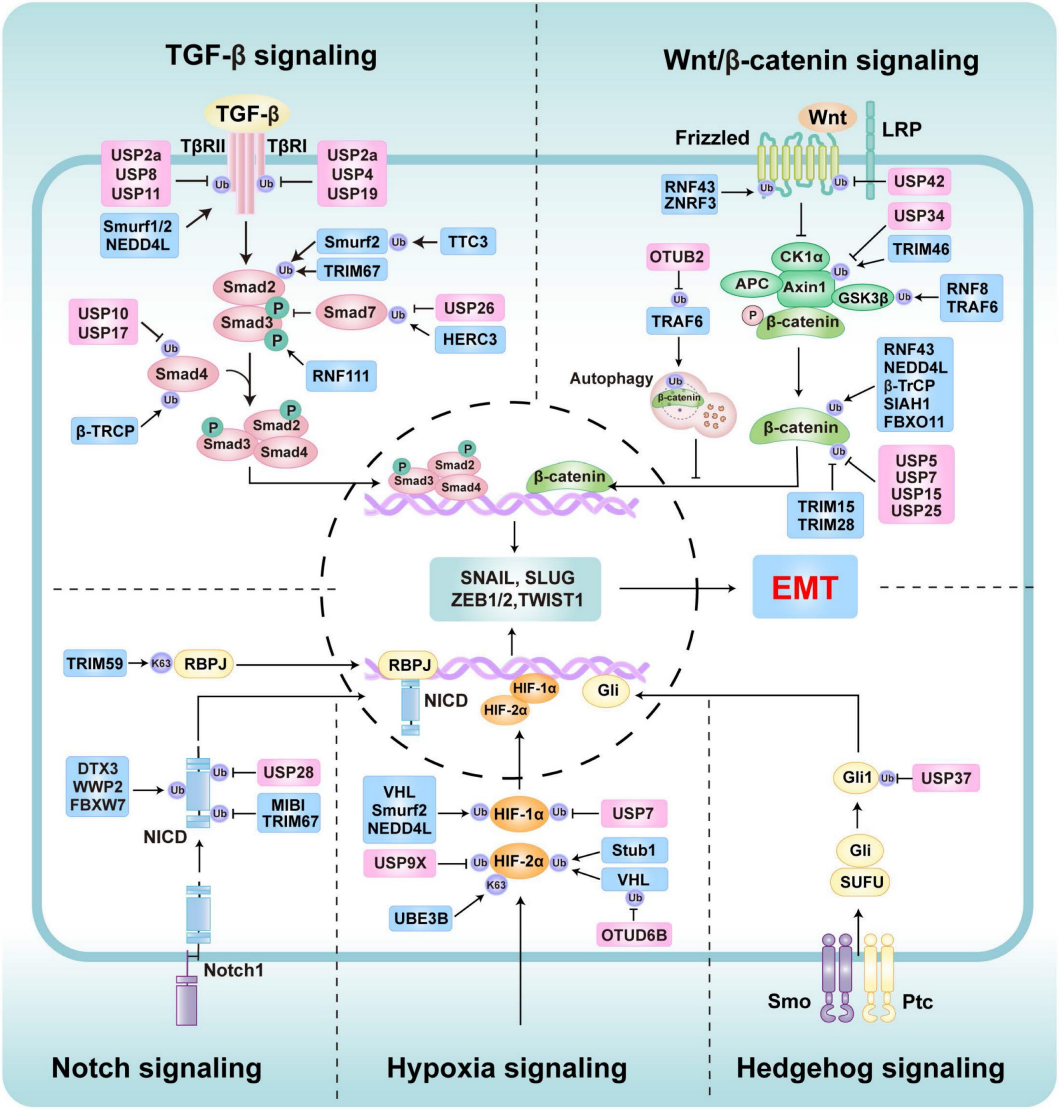
** The role of E3s and DUBs involves key signaling pathways in EMT regulation, including TGF-β signaling, Wnt/β-catenin signaling, Hypoxia signaling, Hedgehog signaling, and Notch signaling.** These pathways modulate the activity of EMT transcription factors through various mechanisms, thereby promoting or inhibiting the EMT process. The E3s are marked with blue icons, and DUBs are marked with pink icons.

Ubiquitination plays a key role in regulating EMT 19. Several E3 ligases (ubiquitination) and DUBs (deubiquitination) critically regulate EMT by modulating core EMT-TFs such as Snail/Slug, ZEB1/2, and Twist1 (Figure 4, Table 1, and Table 2), as well as key EMT-associated signaling networks such as TGF-β, Wnt/β-catenin signaling, Notch, Hh, and hypoxia signaling (Figure 5, Table 1, and Table 2).

### Ubiquitination regulation of EMT-TFs

#### Snail/Slug regulation

Snail and Slug are key EMT-TFs involved in the regulation of EMT by suppressing E-cadherin expression 73. The stability and activity of Snail/Slug are primarily governed by E3 ligases and DUBs. Generally, E3 ligases inhibit EMT by facilitating the ubiquitination and proteasomal degradation of these proteins (Table 1). For instance, SPRY Domain-Containing SOCS Box Protein 3 (SPSB3) promotes Snail degradation in a GSK-3β phosphorylation-dependent manner to limit EMT 74. C-terminus of HSC70-interacting protein (CHIP) ubiquitinates Snail by K48-linked ubiquitin chains, leading to its degradation and inhibiting the EMT process 75, 76. Similarly, MARCH2 directly interacts with Snail to induce its ubiquitination and subsequent proteasomal degradation, thereby suppressing EMT 22. Other E3 ligases, such as MDM2 77, 78, FBXW1/β-TRCP1 79, 80, FBXW7/FBW7 81, FBXL5 82, 83, FBXL14/Ppa 84, 85, FBXO11^86^, FBXO28 87, FBXO31 88, FBXO45 89, TRIM21 90, TRIM50 91, and HECTD1 92 can degrade Snail or Slug to inhibit EMT. F-box proteins play a major role in regulating the functions of Snail and Slug proteins, and are also closely related to tumor metastasis. These E3 ligases are usually downregulated in aggressive cancers, enabling Snail accumulation to promote EMT. However, a few E3 ligases like Pellino-1 93, 94 and RNF8 95 can stabilize Snail or Slug by K63-linked ubiquitin chains to facilitate EMT. Additionally, A20 (TNFAIP3) stabilizes Snail through monoubiquitination, thereby promoting EMT in response to TGF-β1 96.

Conversely, DUBs stabilize Snail or Slug by removing ubiquitin modifications, thereby driving the EMT process (Table 2). For instance, USP1 stabilizes Snail by removing K48-linked polyubiquitin chains, increasing its stability and promoting EMT progression 97. Similarly, DUB3 can also stabilize Snail through deubiquitination, thereby promoting the EMT process 98. Other DUBs such as USP2 99, USP3 100, USP4 101, USP5 21, USP9X 102, USP10 103, USP11 104, USP13 105, USP17 106, USP18 107, USP26 108, USP27X 109, USP28 110, USP29 111, USP30 112, USP35 113, USP37 114, USP41 115, USP47 116, OTUB1 117, OTUD4 118, Eukaryotic translation initiation factor 3 subunit H (EIF3H) 119, Josephin domain-containing 1 (JOSD1) 120, PSMD14 121 enhance the stability of Snail by removing ubiquitin chains to stabilize it, facilitating EMT progression. Furthermore, only a few DUBs like USP5 122, USP10 123, USP20 124, and DUB3 125 can stabilize Slug by deubiquitinating to promote the EMT. Thus, DUBs mainly promote EMT by stabilizing Snail.

#### ZEB1/2 regulation

ZEB1 and ZEB2 act as master transcriptional repressors of EMT, primarily inhibiting E-cadherin expression to disrupt intercellular junctions and initiate EMT 126. They also induce the expression of mesenchymal markers, including vimentin, thereby enhancing cellular migration and invasion capabilities 127. The stability of ZEB1 and ZEB2 is dynamically regulated by ubiquitination and deubiquitination (Table 1 and Table 2). Specific E3 ligases, such as FBXO11, directly ubiquitinate and degrade ZEB1 to inhibit EMT 128. Additional E3 ligases such as TRIM26 129, SIAH 130, FBXO45 89, FBXW7 131, 132, and FBXL14 85, which can also negatively regulate EMT by targeting ZEB1 or ZEB2 for degradation.

DUBs critically regulate ZEB1 stability through deubiquitination to regulate EMT and metastasis. For instance, USP22 stabilizes ZEB1 to activate ZEB1-mediated transcriptional activation and drive EMT 133. Similarly, USP51 stabilizes ZEB1 through deubiquitination, thereby promoting mesenchymal activation and stromal recruitment in gastric cancer (GC) and lung adenocarcinoma (LUAD) 134, 135. Moreover, CDK4/6 further amplifies this process in LUAD by phosphorylating USP51 134. Other DUBs such as USP18 136, USP21 137, USP39 129. USP43 138, and BRCA1-BRCA2-containing complex subunit 3 (BRCC3) 139 enhance the stability of ZEB1 by removing ubiquitin chains, facilitating cell migration and EMT progression. Conversely, USP10 promotes ZEB1 degradation in CRC by removing K27-linked ubiquitin chains, thereby inhibiting EMT 140. These findings underscore the critical dynamic balance between ubiquitination by E3 ligases and deubiquitination by DUBs in controlling ZEB1 stability, thereby regulating the EMT process.

#### Twist1 regulation

Twist1 is another critical transcription factor in regulating EMT by directly binding to the E-box motif to repress E-cadherin expression and activate mesenchymal genes 141. Its stability is mainly regulated through the ubiquitination and deubiquitination processes to control the progression of epithelial-mesenchymal transition (Table 1 and Table 2). For instance, the E3 ligase SPOP (speckle-type POZ protein) ubiquitinates and degrades Twist1 to suppress EMT progression 142. Other E3 ligases like BTRC 143, β-TRCP 144, FBXL14 145, and FBXO45 89 promote the degradation of Twist1 to inhibit EMT. Conversely, E3 ligase RNF8 146, RBX1^147^, and FBXO3 148 stabilize Twist1 to activate EMT and cancer progression. Furthermore, DUBs can stabilize Twist1 to promote EMT. For instance, USP5 stabilizes Twist1 through deubiquitination, thereby activating EMT in bladder cancer 149. Moreover, USP13 similarly stabilizes Twist1 to facilitate EMT 150. Other DUBs like DUB3 125, USP4 148, USP18 151, and USP29 152 can also stabilize Twist1 through deubiquitination, thereby facilitating EMT progression. Conversely, DUB TRAF-binding domain (Trabid) can promote the degradation of Twist1 by removing K63-linked ubiquitin chains, leading to its degradation and EMT inhibition in hepatocellular carcinoma (HCC) 153. Together, E3 ligases and DUBs regulate Twist stability to control EMT progression.

### Ubiquitination Regulation in EMT-Related Signaling Pathways

#### TGF-β signaling regulation

TGF-β signaling is a prominent pathway for the induction of EMT 8. The canonical SMAD pathway involves TGF-β ligands binding to TGF-β type II receptor (TβRII), which then recruits and activates TGF-β type I receptor (TβRI) 52. Activated TβRI phosphorylates SMAD2 and SMAD3, which form complexes with SMAD4 that translocate to the nucleus to regulate EMT-related gene expression 52. However, SMAD7 inhibits this pathway by directly binding TβRI or disrupting SMAD complex formation 52. Ubiquitination critically regulates TGF-β/SMAD-induced EMT by targeting pathway components (Table 1). For instance, Smurf1 directly ubiquitinates TβRII by K48-linked polyubiquitin chains to degrade it, suppressing TGF-β-induced EMT 154. Similarly, Smurf2 promotes proteasomal degradation of SMAD2 and TGFβ receptor, thereby suppressing TGF-β-induced EMT 155-157. Furthermore, other E3 ligases like β-TrCP 158, TRIM67 159, and NEDD4L 160 also negatively regulate TGF-β-induced EMT by SMAD proteins or TGF-β receptors. Conversely, HERC3 promotes the autophagic degradation of Smad7 through K63-linked polyubiquitin chains, thereby enhancing TGF-β/SMAD-induced EMT 161. Intriguingly, another research showed that HERC3 ubiquitinates and degrades EIF5A2, thereby inhibiting the EMT induced by the EIF5A2/TGF-β/Smad2/3 signaling pathway in CRC 162. These two opposite results further demonstrate the complexity and heterogeneity of tumor cell signal regulation. Other E3 ligases, including MDM2 163, RNF61 164, RNF111 165, 166, and TTC3 167, enhance TGF-β signaling and EMT by stabilizing receptors or facilitating signal transduction.

The regulatory balance is further influenced by DUBs, which remove ubiquitination levels to modulate TGF-β/SMAD signaling pathways (Table 2). For example, USP1 stabilizes AK1 to promote TGF-β-induced EMT in TNBC cells 168. USP4 stabilizes TβRI by removing the ubiquitination, thereby accelerating TGF-β1-induced EMT and contributing to renal interstitial fibrosis and HCC 169, 170. Moreover, USP10 and USP17 stabilized SMAD4 through their deubiquitinase activity, thereby enhancing TGF-β SMAD-dependent signaling and promoting EMT in OS and HCC 171, 172. Specifically, USP19 exhibits isoform-dependent functions in regulating TGF-β signaling and EMT. In breast cancer (BC) models, the cytoplasmic isoform USP19 stabilizes both the TβRI and TβRII to enhance TGF-β-induced EMT and cell migration, whereas the endoplasmic reticulum-localized isoform USP19 inhibits EMT by reducing TβRI surface expression 173. Other DUBs, such as USP2a 174, USP3 175, USP8 176, and USP11^177^, drive TGF-β-induced EMT by stabilizing key TGF-β receptors. Conversely, USP26 inhibits this pathway by deubiquitinating and stabilizing SMAD7, preventing formation of the SMAD2/3-TβRI complex and suppressing TGF-β-induced migration and invasion in glioblastoma (GBM) 178. Collectively, this intricate interplay of ubiquitination and deubiquitination mechanisms precisely regulates TGF-β-mediated EMT.

#### Wnt/β-catenin signaling regulation

The Wnt/β-catenin signaling pathway plays a pivotal role in regulating EMT, facilitating the shift from epithelial to mesenchymal characteristics during processes such as cancer metastasis 179, 180. Ubiquitination modulates the Wnt/β-catenin signaling pathway, thereby regulating EMT (Table 1). Signal initiation occurs through the binding of Wnt ligands to Frizzled (FZD) receptors and LRP5/6 co-receptors at the cell surface, which stabilizes β-catenin by inhibiting its degradation complex and preventing ubiquitination-mediated proteasomal targeting 181. Specific E3 ubiquitin ligases such as RNF43/ZNRF3 negatively regulate Wnt signaling by promoting FZD receptor ubiquitination and degradation, thereby suppressing EMT 182. Furthermore, RNF43 inhibits Wnt/β-catenin signaling by downregulating β-catenin, thereby enhancing EMT in TNBC cells 183. Other E3s like β-TrCP 184, FBXO11 185, SIAH1-SIP-Skp1 complex 186, and NEDD4L 187 can also induce ubiquitination and degradation of β-catenin to inhibit Wnt/β-catenin signaling and negatively regulate EMT. Conversely, TNF receptor-associated factor 6 (TRAF6) activates β-catenin signaling and EMT by mediating ubiquitination and degradation of GSK3β 188. However, another study showed that TRAF6 paradoxically inhibits the Wnt pathway by promoting the autophagic degradation of β-catenin, thereby suppressing EMT in CRC 189. Other E3s like TRIM46 promote Wnt/β-catenin signaling by degrading Axin1 to promote hypoxia-induced EMT in HK2 cells 190. Similarly, TRIM15 191, TRIM28 192, and RNF8 193 can also activate Wnt/β-catenin signaling and EMT by stabilizing β-catenin.

Furthermore, DUBs regulate EMT by removing ubiquitin chains to stabilize key components of Wnt/β-catenin signaling (Table 2). For instance, USP34 stabilizes Axin1 through deubiquitination to maintain the integrity of the β-catenin destruction complex, thereby reducing β-catenin levels and inhibiting EMT 194. USP22 deubiquitinates and stabilizes ADAM9 to inhibit Wnt/β-catenin signaling and EMT in trophoblast cells 195. Moreover, USP42 suppresses EMT by stabilizing ZNRF3/RNF43, which promotes FZD receptor ubiquitination and degradation 182. Conversely, USP5 stabilizes β-catenin by removing ubiquitin, driving Wnt/β-catenin signaling-induced EMT 196. Additionally, other DUBs like USP7 197-199, USP13 200, USP15 201, USP25 202, USP36 203, and OTUB2 204 similarly promote the stabilization of β-catenin or other elements, enhancing Wnt/β-catenin signaling to facilitate EMT. Collectively, ubiquitination dynamics serve as a critical molecular switch governing the activity of the Wnt/β-catenin pathway and thereby modulating EMT.

#### Notch signaling regulation

The Notch signaling pathway, an evolutionarily conserved system of intercellular communication, governs diverse cellular processes including differentiation, proliferation, apoptosis, and stem cell self-renewal 205. Substantial evidence demonstrates that Notch signaling modulates EMT through direct transcriptional regulation and intricate crosstalk with pathways such as TGF-β and Wnt/β-catenin 52. E3 ligases critically regulate this process by substrate-specific ubiquitination of Notch signaling components, thereby determining their stability, subcellular localization, and signaling output (Table 1 and Table 2). For instance, Deltex E3 ubiquitin ligase 3 (DTX3) and WW domain containing E3 ubiquitin protein ligase 2 (WWP2) bind to Notch intracellular domain (NICD), promoting its ubiquitination and degradation to suppress Notch-induced EMT 206, 207. Conversely, USP28 stabilizes NICD to activate Notch-induced EMT 208. Furthermore, FBXW7 ubiquitinates and degrades Notch1 to suppress Notch signaling-induced EMT 209, while E3 ligase MIB1 and TRIM67 promote EMT and cell invasion in NSCLC by positively regulating the Notch signaling 210, 211. Under Hypoxia, NICD overexpression causes degradation of ataxin-1 (ATXN1) by MDM2, thereby enhancing Snail expression to induce EMT in cervical cancer cells 212. Other mechanisms involve E3 ligases such as TRIM59 that stabilize recombination signal binding protein for immunoglobulin kappa J region (RBPJ) to activate Notch signaling to induce EMT and metastasis 213.

#### Hypoxia-induced signaling regulation

Hypoxia, a hallmark of solid tumors, critically promotes EMT to enhance cancer cell migration and invasion 214, 215. This induction is primarily mediated by hypoxia-inducible factors (HIFs), a member of key TFs activated under low oxygen conditions 215. HIFs, particularly HIF-1α, directly regulate the expression of EMT-related genes, including Snail, Slug, ZEB1/2, and Twist1/2 216, 217. This regulation occurs through HIF-1α binding to hypoxia response elements in the promoter regions of these genes, driving their expression and promoting EMT. Furthermore, Multiple signaling pathways, including TGF-β, Wnt/β-catenin, PI3K/AKT/mTOR, and Notch, are modulated by hypoxia to further induce EMT 218-221. The E3 ligase von Hippel-Lindau (VHL) is a crucial tumor inhibition factor, which plays a key role in Hypoxia-induced EMT by ubiquitinating and degrading HIFs 222, 223. VHL knockdown in ccRCC leads to HIF-1α/2α accumulation, which upregulates N-cadherin and vimentin while suppressing E-cadherin, ultimately facilitating tumor invasion 224. Moreover, deubiquitylase ovarian tumor domain-containing 6B (OTUD6B) stabilizes VHL to suppress HIF-1α/2α-mediated EMT and metastasis in HCC and ccRCC 225, 226. Other E3 ligases like Smurf2 and NEDD4L also regulate HIF-1α stability to constrain EMT 187, 227. In contrast, E3 ligase UBE3B stabilizes HIF-2α by K63-linked polyubiquitin chains to promote lung metastasis 228. Acute hypoxia induces acetylation of STIP1 homology and U-box containing protein 1 (Stub1), which promotes deubiquitinate of HIF-2α and inhibits EMT-associated vascular remodeling 229. Additional DUBs like USP7 230 and USP9X 231 enhance HIF-1α or HIF-2α signaling by counteracting ubiquitination, thereby indirectly influencing the expression of EMT-TFs (Table 1 and Table 2).

#### Hh signaling regulation

Hh signaling pathway is an evolutionarily conserved mechanism involving key components such as Patched (Ptc), Smoothened (Smo), and Glioblastoma-associated oncogene homolog (Gli) 232. It plays a significant role in EMT by upregulating EMT-TFs, which are critical for cancer metastasis and chemoresistance 233. Gli1 acts as a primary effector with its aberrant activity linked to the Hh-dependent induction of key EMT regulators, including Snail, Slug, and Twist 234. The stability of Gli, Ptc, and Smo is regulated by ubiquitination, which becomes a crucial regulatory mechanism in the Hh signaling pathway 235. For instance, novel E3 ligases like Btbd9 and Kctd3 positively regulate Hh signaling 236, while Smurf1 and Smurf2 suppress the Hh/Gli signaling pathway by ubiquitinating and degrading Gli1 via K48-linked polyubiquitination 237. Despite numerous studies showing that E3 ligases can regulate the Hh signaling by regulating Gli, E3 ligases in the Hh-mediated EMT process remain limited. DUBs like USP37 enhance Gli1 stability and activate Hh signaling by deubiquitinating, facilitating EMT-induced metastasis and chemoresistance in BC cells 238. Similarly, USP5 and USP7 promote Hh signaling by stabilizing Gli1 through deubiquitination 239, 240, and UCHL5/UCH37 stabilizes Smo to enhance Hh signaling 241. However, whether they are involved in regulating EMT in tumors requires further investigation. Consequently, the dysregulation of ubiquitination in the Hh pathway is closely associated with EMT and cancer progression. Targeting ubiquitination-related enzymes may offer new therapeutic strategies for Hh signal-driven EMT and metastasis.

## Ubiquitination-regulated EMT in cancer metastasis

EMT functions as a key driver of tumor metastasis, a complex biological process finely controlled by ubiquitination and deubiquitination 50. Dysregulation of EMT boosts cancer spread by enhancing cell migration, invasion, and resistance to apoptosis 242. This section aims to explore the role of E3 ligases or DUBs-regulated EMT in tumor metastasis and analyze their potential promoting mechanisms (Table 3).

### Stabilizing Snail/Slug and promoting metastasis

Snail and Slug critically regulate tumor metastasis by inducing EMT, a process governed by their modulation of genes involved in cell adhesion, polarity, and cytoskeletal dynamics 243. Under normal conditions, the levels of Snail or Slug are maintained at low steady-state concentrations through proteasomal degradation, ensuring precise control of EMT progression 243. However, dysregulation of ubiquitination often leads to the stabilization of the Snail/Slug proteins, thereby facilitating EMT and metastatic dissemination (Table 3) 244. For instance, the E3 ligase Pellino-1 is abnormally highly expressed in LUAD and TNBC, which often leads to metastasis and a lower survival rate for patients. Mechanistically, the highly expressed Pellino-1 stabilizes Snail or Slug by K63 ubiquitination to promote EMT and metastasis *in vitro* and* in vivo* 93, 94. Similarly, RNF8 also stabilizes Slug via K63 ubiquitination, thereby driving EMT and metastasis in lung cancer cells *in vivo* 95. Furthermore, A20 promotes the monoubiquitination of Snail, thereby facilitating TGF-β1-induced EMT and metastasis in BC cells *in vivo* 96. Since most E3 ligases degrade Snail or Slug through the K48-linked ubiquitin chain, they inhibit the EMT process and tumor metastasis. Therefore, multiple E3 ligases are expressed at low levels in tumors, which facilitates the EMT-induced metastasis. Conversely, DUBs play a crucial role in promoting EMT-induced cancer metastasis.

DUBs inhibit the degradation of the Sanil protein mediated by the K48 ubiquitin chain, thereby promoting EMT and driving tumor metastasis (Table 3). For instance, USP1 97 and USP11 104 stabilize Snail to promote the invasion and metastasis of OC cells *in vitro* and* in vivo*. In choroidal melanoma, USP2 overexpression facilitates EMT migration and invasion by stabilizing Snail *in vitro* 99. Furthermore, USP4 101 and DUB3 98 stabilize Snail and induce EMT, thereby promoting HCC cell migration, invasion, and tumor metastasis *in vitro* and* in vivo*. In bladder cancer and HCC, USP5 deubiquitinates and stabilizes Slug to induce EMT, facilitating cancer cell migration, invasion, and tumor metastasis *in vitro* and* in vivo* 122, 245. Moreover, USP5 21, USP18^107^, and USP47 116 suppress Snail degradation to induce EMT, thereby promoting CRC cell migration and tumor metastasis *in vitro* and* in vivo*. In BC, USP9X 102, USP10 103, USP20 124, USP27X 109, USP28 110, USP30 112, USP41^115^, USP47 246, OTUD4 247, and DUB3 248 are often abnormally highly expressed. Those DUBs stabilize Snail or Slug by removing ubiquitination, thereby increasing EMT and BC cell migration and tumor metastasis *in vitro* and* in vivo*. Moreover, OTUD4 also directly deubiquitinates and stabilizes Snail to promote melanoma cell migration and tumor metastasis *in vitro* and *in vivo* 118*.* Similarly, USP13 105, USP29 111, USP35 113, and USP37 249 stabilize Snail to repress E-cadherin, enhancing epithelial-mesenchymal plasticity and driving GC cell migration and tumor metastasis *in vitro* and* in vivo*. In oral squamous cell carcinoma cells (OSCC), USP17 promotes the stability of Snail, leading to migration and invasion involving EMT *in vitro* 106. Furthermore, USP26 108, PSMD14 121, OTUB1 117, and EIF3H 119 deubiquitinate and stabilize Snail, enhancing esophageal squamous cell carcinoma (ESCC) cell migration and tumor metastasis *in vivo* and *in vitro*. In LUAD, USP37 114 and JOSD1 120 deubiquitinate Snai1 to activate EMT, thereby promoting cell invasion and migration *in vitro*. Therefore, the abnormal expression of these DUBs in tumors is a key factor that promotes the stability of Snail and tumor metastasis.

### Stabilizing ZEB1 and promoting metastasis

ZEB1 promotes cell migration and invasion by regulating cytoskeletal remodeling, cell-cell adhesion, and increasing the expression of vimentin 250. Several DUBs stabilize ZEB proteins through deubiquitination, thereby enhancing EMT and tumor metastasis (Table 3). For instance, USP21 and USP43 deubiquitinate and stabilize ZEB1 to induce EMT, enhancing cell migration and stemness in CRC *in vitro* 137, 138. Similarly, USP18 stabilizes ZEB1 by deubiquitinating to facilitate ESCC cell migration and tumor metastasis *in vitro* and* in vivo* 136. USP22 stabilizes ZEB1 to induce angiogenesis and EMT, thereby promoting the invasion and migration of OC *in vitro* 133. In HCC and multiple myeloma (MM) cells, USP39 stabilizes ZEB1 by deubiquitination to induce EMT, thereby promoting cell migration and tumor metastasis *in vitro* and *in vivo* zebrafish experiments 129, 251. Similarly, USP51 stabilizes ZEB1 through deubiquitination, promoting metastatic dissemination in GC *in vitro* and *in vivo* 135. Further research showed that CDK4/6 phosphorylation of USP51 is required for ZEB1-mediated metastasis in LUAD and BC *in vitro* and *in vivo* 134, 252. Concurrently, BRCC3 stabilizes ZEB1 by deubiquitination to induce EMT, thereby promoting TNBC cell migration, invasion, and tumor metastasis *in vitro* and *in vivo* 139. However, USP10 degrades ZEB1 by removing K27-linked ubiquitin chains, thereby suppressing CRC cell migration mediated by ZEB1 *in vitro* 140. Conversely, ERK-mediated phosphorylation of USP10 at Ser236 impairs its interaction with ZEB1, thereby stabilizing ZEB1 and promoting CRC metastasis *in vivo* 140. Together, these mechanisms illustrate how the interplay between E3 ligases and DUBs regulates ZEB stability to drive cancer progression.

### Stabilizing Twist1 and promoting metastasis

Twist1 represses E-cadherin transcription, promoting tumor cell migration, invasion, and metastasis 243. Clinically, elevated Twist1 correlates with increased lymph node metastasis, distant metastasis, and advanced tumor stage across multiple cancers 253. The protein stability of Twist1 is dynamically regulated by E3s and DUBs, which promote EMT and tumor metastasis by stabilizing Twist1(Table 3). For instance, USP13 directly interacts with Twist1 and cleaves FBXL14-induced K48-linked polyubiquitin chains, increasing Twist1 protein levels and facilitating BC cell migration and tumor metastasis *in vitro* and *in vivo* 150. In GBM, USP18 deubiquitinates and stabilizes Twist1, thereby inducing EMT and promoting cell migration and tumor metastasis in* vitro* and *in vivo* 151. Similarly, USP29 stabilizes Twist1 through deubiquitination, thereby driving malignant phenotypes in TNBC *in vitro* and *in vivo* 152. Furthermore, specific E3 ligases can also stabilize Twist1 to facilitate metastasis. For instance, RNF8 can stabilize Twist1 to induce EMT through K63-linked ubiquitin chains, thereby enhancing BC cell migration, invasion, and tumor metastasis *in vitro* and* in vivo* 146*.* FBXO3 disrupts the DNPEP-mediated degradation of USP4, stabilizing Twist1 and promoting BC cell migration and tumor metastasis *in vitro* and* in vivo* 148. RBX1 ubiquitinates and degrades FBXO45, consequently stabilizing Twist1to drive EMT and facilitating TNBC cell invasion, migration, and tumor metastasis *in vitro* and *in vivo* 147. Additionally, USP4 254 and USP51 255 stabilize Twist1 polyubiquitination, enhancing EMT-associated stemness and malignancy in lung cancer *in vitro*. This regulatory network highlights Twist1 as a critical convergence point for post-translational modifications that orchestrate EMT and metastatic progression.

### Activating TGF-β signaling to drive EMT-induced metastasis

TGF-β-induced EMT is a pivotal factor for tumor metastasis, involving multiple EMT-TFs and EMT signaling pathways 256. E3 ligases and DUBs act as key regulators by modulating the stability and activity of TGF-β pathway components, thereby influencing EMT and tumor metastasis (Table 3). For instance, E3 ligase RNF61 degrades Smad nuclear-interacting protein 1 (SNIP1) to activate TGF-β-mediated EMT, promoting CRC cell invasion and tumor metastasis *in vitro* and *in vivo* 164. Furthermore, HERC3 promotes the autophagic degradation of Smad7 through ubiquitination, thereby activating the TGF-β signaling and driving EMT and GBM cell invasion and tumor metastasis *in vitro* and *in vivo* 161. In OC, MDM2 promotes EMT by activating the TGF-β-Smads-Snail/Slug pathway, enhancing OC cell invasion and migration *in vitro* 163. Similarly, RNF111 is highly expressed in the high-metastatic NSCLC cell line 95D, which activates TGF-β signaling-induced EMT to enhance NSCLC cell invasion and migration *in vitro* 166. Furthermore, DUBs stabilize core TGF-β signaling proteins by removing ubiquitin chains, thereby promoting EMT and tumor metastasis. For instance, the USP1/WDR48 complex stabilizes TAK1 through deubiquitination to enhance EMT and cell migration in TNBC *in vitro* 168. In lung cancer, USP2a stabilizes TβRI by removing K33-linked polyubiquitin chains to promote nuclear translocation of SMAD2/3, activating TGF-β-induced EMT and metastasis *in vivo* 174. Similarly, USP4 and USP10 activate the TGF-β signaling through deubiquitinating TβRI and Smad4, thereby promoting EMT-induced cell migration, invasion, and metastasis of HCC *in vitro* and *in vivo* 170, 172. Other mechanisms involve USP3 interacts with and stabilizes SUZ12 by deubiquitination, promoting TGF-β1-induced EMT and cell migration and invasion in GC *in vitro* 175. Furthermore, USP8 176 and USP11 177 enhance TGF-β/SMAD signaling by deubiquitinating and stabilizing TβRII, increasing plasma membrane expression and promoting EMT, invasion, and metastasis in BC cells *in vitro* and *in vivo*. USP17 promotes TGF-β-induced EMT by stabilizing SMAD4, thereby promoting OS cell migration and invasion *in vitro* 171. Specifically, the cytoplasmic isoform USP19 expression is higher in BC tissues and is correlated with poor prognosis. Mechanistically, cytoplasmic isoform USP19 stabilizes TβRI and TβRII to enhance TGF-β-induced EMT and BC cell migration and extravasation *in vitro* 173. Conversely, endoplasmic reticulum-localized isoform USP19 inhibits BC cell migration 173. Therefore, the ubiquitination-related factors play a significant role in promoting tumor metastasis through TGF-β-mediated EMT.

### Activating Wnt/β-catenin signaling to drive EMT-induced metastasis

The dysregulation of Wnt/β-catenin signaling promotes EMT and tumor metastasis across various malignancies through ubiquitination and deubiquitination events that regulate β-catenin stability (Table 3) 257, 258. Emerging evidence showed that RNF8 is overexpressed in highly metastatic BC cell lines. It activates β-catenin-induced EMT by inactivating GSK-3β, thereby promoting BC cell migration and tumor metastasis *in vitro* and *in vivo* 193. Similarly, the non-SMC concentrate I complex subunit (NCAPG) stabilizes β-catenin through competitive binding with SIP to inhibit SIAH1 activity, promoting β-catenin-induced EMT and HCC cell migration and tumor metastasis *in vitro* and *in vivo* 186. Furthermore, elevated expression of TRIM15 in ESCC tissues and cell lines activates Wnt/β-catenin-induced EMT, leading to cell migration, invasion, and tumor metastasis *in vitro* and *in vivo* 191. Additionally, TRIM28 has been implicated in OC cell metastasis *in vitro*, as its knockdown significantly attenuates Wnt/β-catenin signaling and suppresses EMT processes 192.

DUBs can stabilize these key components through deubiquitination, thereby enhancing Wnt/β-catenin signaling and tumor metastasis. For instance, USP5 stabilizes β-catenin to activate the Wnt/β‑catenin and EMT, thereby promoting NSCLC cell migration and invasion *in vitro* 196. Similarly, USP7 activates Wnt/β‑catenin-induced EMT by stabilizing β-catenin, thereby enhancing OS cell migration and invasion *in vitro* 198. Furthermore, in CRC, USP7 augments Wnt/β-catenin signaling by stabilizing DDX3X, promoting EMT and cell migration *in vitro* 197. Similarly, USP13 stabilizes WISP1 to promote the Wnt/β-catenin-induced EMT, driving ESCC cell migration and tumor metastasis *in vitro* and *in vivo* 200. USP15-mediated β-catenin stabilization facilitates EMT and GC cell invasion *in vitro* 201. In HCC, the USP25-TRIM21 axis activates β-catenin signaling-induced EMT and drives cell migration, invasion, and tumor metastasis *in vitro* and *in vivo* 202. Furthermore, OTUB2 stabilizes β-catenin by suppressing TRAF6-mediated autophagy-dependent degradation and activating Wnt/β-catenin-induced EMT, thereby driving intrahepatic cholangiocarcinoma (iCCA) cell invasion and tumor metastasis *in vitro* and *in vivo* 204. These findings demonstrate that ubiquitination-mediated regulation of Wnt/β-catenin signaling constitutes a critical mechanistic node controlling EMT and metastasis across diverse malignancies.

## Ubiquitination-regulated EMT in chemoresistance and strategies

### Ubiquitination-regulated EMT in chemoresistance

Chemotherapy resistance is a major challenge in cancer treatment 259. The resistance mechanism often involves EMT activation, which causes cancer cells to gain stem cell-like qualities, increased migration ability, and decreased sensitivity to chemotherapy 260. Emerging evidence shows that the development of chemoresistance in cancer therapy is frequently connected to ubiquitination-regulated EMT 50. In this section, we aim to explore the role of E3 ligases or DUBs-regulated EMT in tumor chemoresistance and highlight that E3 ligases or DUBs inhibitors are crucial for overcoming tumor metastasis and chemoresistance (Table 4) 232-234.

#### Cisplatin

Platinum-based chemotherapy is a primary treatment for solid tumors, but its efficacy is frequently undermined by the development of drug resistance 261. Cisplatin, a DNA-damaging agent and widely utilized in NSCLC, often encounters resistance mediated by ubiquitin-regulated EMT (Table 4) 262. For instance, the lipid metabolism enzyme carnitine palmitoyltransferase 1C (CPT1C), highly expressed in NSCLC cells, contributes to cisplatin resistance by inducing EMT *in vitro* 263. Mechanistically, cisplatin treatment induces the degradation of the E3 ligase NEDD4L, leading to enhanced CPT1C stability and subsequent EMT-driven cisplatin resistance 263. Intriguingly, NEDD4 exhibits context-dependent roles. In cisplatin-resistant nasopharyngeal carcinoma (NPC) cells, NEDD4 expression contributes to EMT, and its downregulation reverses resistance *in vitro* 264. Similarly, the E3 ligase FBXW7 suppresses EMT and chemoresistance in NSCLC by degrading Snail, whereas reduced FBXW7 expression in patient tissues correlates with poorer treatment response *in vitro* 265. Conversely, the downregulation of cyclin D3 in cisplatin-resistant LUAD cells impairs PARK2-mediated vimentin degradation, stabilizing vimentin and promoting EMT and chemoresistance *in vitro* and *in vivo* 266. Other E3 ligases contribute to EMT-induced resistance: TRAF6 mediates the ubiquitination and degradation of GSK3β, activating β-catenin signaling to promote EMT and cisplatin resistance *in vitro* and *in vivo* 188. Furthermore, Hakai stabilizes phosphorylated AKT to enhance EMT, leading to cisplatin resistance in NSCLC *in vitro* 267.

DUBs significantly facilitate EMT to promote cisplatin resistance in various cancers. For instance, USP1 stabilizes Snail through deubiquitination after platinum-based treatments, thereby inducing EMT and conferring resistance in OC cells *in vitro* 97. Similarly, USP9X enhances Snail stability by removing K48-linked ubiquitin chains, contributing to cisplatin and doxorubicin (Dox) resistance in TNBC *in vitro* and *in vivo* 102, while TGF-β-induced USP27X stabilizes Snail and activates cancer-associated fibroblasts, reducing cisplatin sensitivity in TNBC *in vitro* and *in vivo* 109. Additionally, PSMD14 stabilizes Snail by deubiquitination to drive EMT and diminish cisplatin efficacy in ESCC cells *in vitro* and *in vivo* 121, 268. Notably, key EMT-TFs like ZEB1 and Twist directly promote cisplatin resistance by enabling EMT-linked chemoresistance. For instance, USP51 stabilizes ZEB1 to promote A549 cells' cisplatin resistance *in vitro* 269, and USP29 stabilizes Twist1 to enhance EMT, metastasis, and cisplatin resistance in TNBC *in vitro* and *in vivo* 152. DUBs also regulate TGF-β signaling components, such as USP32 stabilizes SMAD2 to activate TGF-β-mediated proliferation and migration, augmenting cisplatin resistance in GC *in vitro* and *in vivo* 270. In LUAD, USP7 suppresses c-Myc degradation to promote EMT and cisplatin resistance *in vitro* 271, and USP22 stabilizes c-Myc and ALDH1A3 to facilitate EMT and resistance in TNBC and lung cancer *in vitro* and *in vivo* 272, 273. Furthermore, USP37 stabilizes Gli-1 to activate Hh signaling, driving EMT and cisplatin resistance in BC cells *in vitro* and *in vivo* 238. Collectively, DUBs and E3 ligases converge on EMT regulation, establishing them as key mediators of cisplatin resistance and promising therapeutic targets.

#### Oxaliplatin

Oxaliplatin is a standard chemotherapeutic agent primarily employed in the treatment of CRC 274. The resistance of Oxaliplatin is largely through dysregulation of ubiquitin-mediated pathways involving EMT, which play a critical role in tumor metastasis and chemoresistance (Table 4). For instance, OTUB2 stabilizes transcription factor SP1 by removing K48-linked ubiquitin ligases, enhancing EMT and oxaliplatin resistance in CRC *in vitro* and *in vivo* 275. Furthermore, FBXW7 suppresses EMT and chemoresistance in NSCLC by degrading ZEB2, while FBXW7 deletion promotes EMT by enhancing the stabilization of ZEB2, thereby promoting oxaliplatin and 5-fluorouracil (5-FU) resistance in CRC *in vitro*,* ex vivo,* and *in vivo* 132. Therefore, the dysregulation of ubiquitination leads to EMT, which significantly promotes the occurrence of Oxaliplatin resistance.

#### Doxorubicin

Dox, an anthracycline antibiotic, is widely used in cancer chemotherapy for its broad-spectrum activity against various malignancies 276. However, acquired resistance often develops, with ubiquitination pathways playing a crucial role by regulating EMT to confer resistance in different types of cancers (Table 4) 277. In Dox-resistant HCC (HCC/Dox) cells, elevated ZEB1 expression drives EMT-mediated resistance 278. Mechanistically, SIAH1 downregulation inhibits ubiquitination-mediated degradation of ZEB1, thereby enhancing ZEB1 stability to promote chemoresistance in HCC *in vitro* 278. Similarly, the Dox resistance of OS cells is attributed to SIAH1 downregulation, which reduces ZEB1 ubiquitination and stabilizes ZEB1, thereby facilitating EMT and Dox resistance *in vitro* 279. Furthermore, RNF8 stabilizes Twist via K63-linked polyubiquitin chains, promoting nuclear translocation and Dox resistance in TNBC *in vitro* and *in vivo* 146. Conversely, FBXW7 suppresses EMT in HCC cells to enhance Dox sensitivity *in vitro* 280. Further study showed that MiR-223 targets FBXW7 to enhance Dox resistance in CRC *in vivo* 281. Additionally, DUBs augment Dox resistance in various tumors. For instance, USP14 modulates Wnt signaling to enhance resistance in MM *in vitro* 282, and USP29 stabilizes Snail through deubiquitination to boost resistance to Dox and paclitaxel in NSCLC cells *in vitro* and *in vivo* 283. Moreover, USP45 stabilizes MYC in cervical cancer, upregulating vimentin, N-cadherin, and cancer stem cell protein expression to confer Dox resistance *in vivo* 284. These findings collectively showed that ubiquitin-mediated regulation of EMT regulators constitutes a fundamental molecular mechanism for acquiring Dox resistance.

#### Gemcitabine

Gemcitabine (GEM) is the first-line chemotherapy for pancreatic cancer (PC), but its efficacy is frequently compromised by acquired resistance 285. Ubiquitin-mediated EMT is emerging as a critical mechanism underpinning this phenomenon 286. Key ubiquitin-related regulators contribute to this resistance (Table 4). The ubiquitin-protein ligase E3 module N-recognition 5 (UBR5) is markedly upregulated in GEM-resistant PC cells and clinical samples, where it promotes resistance through EMT activation 286. Mechanistically, UBR5 degrades O-GlcNAcase (OGA) by K48-linked ubiquitin chains, thereby inducing EMT and GEM resistance *in vitro* and *in vivo* 286. Concurrently, TRIM59 stabilizes RBPJ by K63-linked ubiquitin chains, activating Notch signaling to induce EMT and drive GEM resistance in PC *in vitro* and *in vivo* 213. RNF126 ubiquitinates and degrades PTEN, thereby activating the AKT/GSK-3β/β-catenin pathway to induce EMT and drive GEM resistance in PC *in vitro* and *in vivo* 287. Moreover, the ubiquitin-like protein FAT10 stabilizes forkhead box protein M1 (FOXM1) by inhibiting ubiquitin-mediated degradation, thereby facilitating EMT and GEM resistance in PC *in vitro* 288. Additionally, microRNA participates in this resistance network, such as miR-15b downregulates Smurf2 to inhibit Smad2/3 ubiquitination and degradation, thereby activating the TGF-β-induced EMT and driving resistance in PC *in vitro* 289, while MiR-223 downregulates FBW7 to exacerbate EMT and resistance in GEM-resistant PC *in vitro* 209. Collectively, these mechanisms highlight ubiquitin-regulated EMT as a pivotal driver of GEM resistance in PC.

#### Others

Beyond mentioned above, multiple therapeutic agents exhibit resistance mechanisms linked to ubiquitin-mediated EMT processes, including temozolomide (TMZ), sorafenib, and Lenvatinib (Table 4) 290, 291. TMZ, a chemotherapeutic alkylating agent commonly used for GBM treatment, is limited in efficacy by resistance mechanisms and the blood-brain barrier despite its first-line status 292, 293. Dysregulation of ubiquitination enhances TGF-β signaling, which contributes to tumor progression and TMZ resistance. For instance, the E3 ligase HERC3 degrades SMAD7 by K48-linked ubiquitin ligase, thereby activating TGF-β signaling, inducing EMT and autophagy, and conferring TMZ resistance in GBM *in vitro* and *in vivo* 161. In HCC, RNF8 promotes EMT and increases sorafenib and Lenvatinib resistance; its silencing suppresses EMT and enhances drug sensitivity by downregulating N-cadherin and Snail *in vitro* 294. Similarly, STAM Binding Protein Like 1 (STAMBPL1) mitigates TRIM21-mediated lysosomal degradation of AXL, reinforcing the mesenchymal phenotype and sunitinib resistance in Kidney Renal Clear cell carcinoma (KIRC) *in vitro* and *in vivo* 295. In BC, USP30 stabilizes Snail through removing K48-linked polyubiquitin chains, accelerating EMT and promoting paclitaxel chemosensitivity *in vitro* and *in vivo* 112. Similarly, OTUD4 stabilizes Snail via ubiquitination to promote malignant phenotypes, driving resistance to BRAF inhibitors like vemurafenib and PLX4720 in melanoma *in vitro* 118. Collectively, ubiquitination and deubiquitination are critical regulators of EMT-associated therapeutic resistance in human malignancies.

### Targeting ubiquitination reverses EMT-induced metastasis and chemoresistance

Targeting ubiquitination-related factors is a prominent research direction in cancer therapy, particularly for countering EMT-mediated metastasis and drug resistance 296. Multiple inhibitors specific to E3 ligases or DUBs effectively reverse these processes 297. For instance, PSMD14 inhibitor Thiolutin reduces Snail stability to inhibit EMT, suppressing motility and stemness while enhancing cisplatin sensitivity in ESCC *in vitro* and *in vivo* 268. Similarly, the Hakai inhibitor Hakin-1 impedes E-cadherin ubiquitination to hinder EMT and tumor progression in CRC *in vitro* and *in vivo* 298. The MDM2 antagonist Nutlin-3 suppresses TGF-β-Smads-mediated EMT and metastasis in OC *in vitro* 163. DUB inhibitors like WP1130 promote Snail degradation by targeting Dub3 or USP9X, inhibiting EMT and metastasis in BC while sensitizing TNBC to cisplatin and paclitaxel *in vitro* and *in vivo* 102, 248. Other examples include Nucleoredoxin interacts with DUB3 to promote Snail degradation via the ubiquitin-proteasome system and suppress HCC progression *in vitro* and *in vivo* 98. Natural compounds such as Erianin from *Dendrobium chrysotoxum* induce Snail degradation via OTUB1 targeting to suppress metastasis in ESCC models *in vitro* 299. Biochanin A facilitates ZEB1 ubiquitination and degradation, reversing EMT-associated cisplatin resistance in LUAD *in vitro* and *in vivo* 300. Cinobufotalin inhibits USP7-mediated MYC deubiquitination to suppress EMT and increase cisplatin sensitivity in LUAD 301, while P5091 reduces USP47-induced EMT in breast epithelial cells 246. Other small molecules like compound 3d disrupt PELI1-Snail/Slug interactions to inhibit EMT and metastasis in TNBC *in vitro* and *in vivo* 302. Similarly, the USP2 inhibitor ML364 induces Snail degradation to inhibit proliferation and metastasis in choroidal melanoma *in vitro* and *in vivo* 99. The USP4 inhibitor U4-I05 degrades β-catenin and Twist1, inhibiting metastasis and enhancing sensitivity to oxaliplatin and 5-FU in CRC *in vitro* and *in vivo* 303. Additionally, the USP28 inhibitor compound 19, a 1,2,3 triazolo [4,5-d] pyrimidine derivative, blocks proliferation and EMT in GC 304. Collectively, these research results indicate that targeting ubiquitination-related factors possesses great potential in addressing EMT-driven tumor metastasis and chemoresistance.

## Drug development targeting E3 ligases and DUBs

### Drug development targeting E3 ligases

E3 ligases act as key regulators by mediating the ubiquitination of specific substrates, ultimately degrading the substrates or enabling signal transmission 305. The dysregulation of E3 ligases is associated with several human diseases, particularly cancers, making them attractive targets for the development of new drugs 305. In this section, we systematically summarize the current situation of drug development targeting E3 ligases and DUBs and their potential mechanisms (Table 5).

#### PROTACs and Molecular Glue Degraders

E3 ligases play a crucial role in the ubiquitination process and are therefore essential components of PROTACs and molecular glues 306, 307. Proteolysis-targeting chimera (PROTAC) technology plays a pivotal role in the development of small-molecule drugs, leveraging the ubiquitin-proteasome system to induce protein degradation 308. PROTACs are heterobifunctional molecules that facilitate the proximity between a target protein and an E3 ligase, leading to ubiquitination and subsequent proteasomal degradation of the target protein 309. Several PROTACs are currently undergoing clinical trials for cancer therapy. For instance, ARV-110, also known as bavdegalutamide, is an investigational drug being evaluated in phase 1/2 clinical trials for the treatment of metastatic castration-resistant prostate cancer (mCRPC) 310. A phase 1b study is also evaluating the combination of ARV-110 with abiraterone in patients with metastatic prostate cancer 311. Furthermore, preclinical studies have demonstrated that ARV-110 can degrade AR by ≥95% and exhibits antitumor activity in enzalutamide-naive and resistant prostate cancer xenograft models 312. ARV-471 is an orally bioavailable PROTAC degrader, which has potential antitumor activity in BC treatment by targeting estrogen receptor (ER) 313. For instance, ARV-471 therapy was well-tolerated and showed antitumor activity in patients with ER+/HER2- locally advanced or metastatic BC in a phase I clinical trial 313; current phase III trials are evaluating its utility in locally advanced and metastatic BC 314. Despite PROTACs having shown great potential, challenges remain in their clinical development, including poor oral bioavailability, large molecular weights, and dependencies on specific E3 ligase receptors 315.

Molecular glues offer an alternative approach to targeted protein degradation 307. These small molecules induce interactions between a target protein and an E3 ligase, leading to the degradation of the target protein 307. Compared to PROTAC, molecular glues have several advantages, including smaller molecules, improved cell permeability, and tissue specificity, enabling more efficient promotion of E3 ligase-target protein interactions and ubiquitination 308, 316. Several molecular glue degraders (MGDs) targeting E3 ligases are currently being evaluated in preclinical and clinical studies for cancer therapy 317. For instance, CC-90009 recruits the cullin4-RING E3 ubiquitin ligase (CRL4)-cereblon (CRBN) complex to induce proteasomal degradation of G1-to-S phase transition 1 (GSPT1), leading to potent suppression of acute myeloid leukemia (AML) in preclinical studies 318. Furthermore, the clinical trial of CC-90009 to treat leukemia is under investigation 318. Overall, targeted protein degradation using PROTACs and molecular glues is a promising therapeutic strategy for treating various diseases, including cancer 319. While challenges remain in their clinical development, ongoing research is focused on improving their selectivity, bioavailability, and efficacy.

#### MDM2 inhibitors

MDM2 inhibitors represent a significant therapeutic approach targeting MDM2, an E3 ligase that negatively regulates p53 activity 320. These inhibitors aim to restore p53 function in tumors harboring wild-type TP53 by disrupting he interaction between MDM2 and p53 321. Several MDM2 inhibitors have progressed to clinical trials, evaluating their safety, efficacy, and optimal dosing across various cancer types 322, 323. Clinical trials primarily focus on patients with cancers retaining wild-type TP53, as the mechanism of action depends on p53 reactivation. MDM2 inhibitors have shown some clinical activity, especially in hematological malignancies such as AML and chronic lymphocytic leukemia (CLL) 321. For instance, a phase I study of RG7112 in hematologic malignancies assessed dose and safety 324, and subsequent phase Ib trials explored RG7112 in combination with Ara-C for AML treatment 325, as well as monotherapy for relapsed/refractory solid tumors 326. Another phase Ib study evaluated RG7112 with Dox in advanced soft tissue sarcoma 327. APG-115, a potent small-molecule MDM2 inhibitor and immune modulator, demonstrated promising antitumor activity 328. Subsequent phase I trials in patients with advanced solid tumors assessed its safety, pharmacokinetics, pharmacodynamics, and antitumor effects 328. A Chinese study (CTR20170975) similarly focused on APG-115 in advanced soft tissue sarcomas 329. Further research suggests that APG-115 enhances programmed death ligand 1 (PD-L1) immunotherapy efficacy in thyroid cancer 330. Additionally, other small-molecule MDM2 inhibitors, including JNJ-26854165 (Serdemetan), ALRN-6924, RG7388 (Idasanutlin), MK-8242, SAR-405838, CGM097, DS3032b (Milademetan), Siremadlin (HDM-201), AMG232, RO6839921, and BI907828, are under investigation in clinical trials to assess their therapeutic potential (Table 5) 321, 331-341. However, resistance to MDM2 inhibitors represents a significant obstacle. The mechanisms of resistance can be categorized broadly as p53-dependent or p53-independent 342. P53-dependent resistance involves alterations in the p53 pathway, such as TP53 mutations or dysregulation of p53 target genes 343. P53-independent resistance entails activation of alternative signaling pathways that circumvent p53 reactivation 344. Furthermore, combination therapies pairing MDM2 inhibitors with chemotherapy, targeted therapies, or immunotherapy offer a promising avenue to enhance efficacy 345. Understanding these mechanisms is critical for developing strategies to overcome resistance.

#### IAP inhibitors

Inhibitor of Apoptosis Proteins (IAPs) are key regulators of programmed cell death, and their inhibitors are emerging as potential cancer therapeutics. Small-molecule IAP inhibitors mimic the endogenous IAP antagonist Smac/DIABLO. They bind with high affinity to cellular IAP1 (cIAP1), cIAP2, and X-linked IAP (XIAP), subsequently inducing their proteasome-dependent degradation 346. Specifically, the IAP antagonist GDC-0152 entered phase I clinical trials to evaluate safety, tolerability, and pharmacokinetics in patients with advanced solid tumors 347, 348. Preclinical studies demonstrate that GDC-0152 binds the XIAP BIR3 domain and the BIR domains of cIAP1/cIAP2, promotes cIAP1 degradation, and reduces the viability of BC cells 346, 347. GDC-0152 can also modulate ABCB1-ATPase activity and suppress BIRC5 expression, mechanisms associated with overcoming multidrug resistance 348. LCL161, another Smac mimetic, has undergone evaluation as a single agent and in combination therapies 349, 350. For instance, LCL161 has been investigated in patients with intermediate or high-risk myelofibrosis who have failed or are intolerant to JAK inhibitors 351. A phase I study in Japanese patients with advanced solid tumors combined LCL161 with paclitaxel, while data suggest an increased risk of infection with the combination 349. Currently, several IAP inhibitors are in clinical trials, including AT-406 (NCT04122625, NCT03871959, NCT02022098, NCT03270176, NCT01078649), TL-32711(NCT01940172), APG-1387 (NCT03386526), AEG40826 (NCT00708006), and BI-891065 (NCT04138823, NCT03166631), demonstrating promising antitumor activity (Table 5) 29, 352-354. Despite their promise, challenges remain with IAP inhibitor development, including toxicity/tolerability issues, optimal patient selection strategies, primary and acquired resistance mechanisms, and potential off-target effects 355. Further research is essential to fully realize the clinical potential of targeting IAPs in cancer therapy.

### Drug development targeting DUBs

DUBs play crucial roles in cellular processes such as protein homeostasis, DNA repair, signal transduction, and epigenetic regulation. Dysregulation of DUB activity is implicated in diverse pathologies, including cancers, autoimmune disorders, chronic inflammation, and neurodegenerative diseases 356. Moreover, DUBs possess well-defined catalytic sites, most of which contain a catalytic cysteine. Consequently, DUBs are emerging as promising targets for drug discovery 357. DUB inhibitors can promote the degradation of oncogenic proteins, particularly those stabilized by DUBs and resistant to direct targeting 356. Specific DUBs, such as USP7, have been identified as potential targets in malignancies, including hematological cancers 358. Based on the types of target enzymes they act upon, the current small molecule inhibitors of DUBs are classified as: USP family inhibitors, UCH family inhibitors, JAMM family inhibitors, MJD family inhibitors, OUT family inhibitors, and SENP family inhibitors 35. Among them, the USP family is the most extensively studied in preclinical research. For instance, HBX19818 and P22077 (USP inhibitors targeting USP7, USP14, and USP22) suppress cancer cell proliferation and enhance the efficacy of conventional therapies like Dox 359, 360. Additionally, targeting DUBs can reverse chemoresistance in cancer cells, offering a strategy for more effective treatment 361, 362. Despite growing interest in DUB biological function and potential as therapeutic targets, few selective small-molecule inhibitors and no approved drugs currently exist 363.

Several small molecules targeting oncogenic DUBs have been identified, with some demonstrating promising anticancer activity and advancing into clinical trials (Table 5) 364, 365. VLX1570, a selective inhibitor of USP14 and UCHL5 derived from b-AP15, exhibited significant antitumor effects in murine models of Waldenstrom macroglobulinemia (WM) by modulating BCR signaling and CXCR4 expression 366. However, its clinical development encountered challenges. In a phase I/II clinical trial (NCT02372240), dose-limiting toxicities were observed in patients with MM during dose escalation, leading to trial termination in 2017 367. Perifosine, an oral alkylphospholipid that targets UCHL3, shows significant activity against BC cells both* in vitro* and *in vivo* 368. In a phase I clinical trial of recurrent/refractory pediatric solid tumors, the Perifosine combination of AKT and mTOR inhibitors was safe and feasible 369. However, in a phase II clinical trial of patients with metastatic BC, only 19% of the patients experienced stable disease after 2 months of treatment 368. KSQ-4279, an inhibitor of USP1, displays strong synergistic activity with PARP inhibitors in BRCA-mutant cancers 370. In a phase 1 trial (NCT05240898), KSQ-4279 showed promising efficacy and safety both as monotherapy and in combination with Olaparib or Carboplatin. Additionally, other USP1 inhibitors such as TNG348, XL309, SIM0501, and HSK39775 are also progressing through clinical trials 35, 364. Notably, TNG348 development was halted due to significant liver toxicity observed in patients treated beyond eight weeks, leading to termination of its phase 1/2 trial (NCT06065059). XL309 has demonstrated preclinical efficacy in BRCA1-mutated TNBC and is being evaluated in an ongoing phase 1 trial (NCT05932862) for safety and preliminary antitumor activity. Furthermore, HSK39775 and SIM0501 are under investigation in phase 1 trials (NCT06331559, NCT06314373) to assess safety and initial efficacy in advanced solid tumors. Collectively, these findings underscore the therapeutic potential of DUB-targeting agents while highlighting critical challenges in development, particularly the need for rigorous safety monitoring during early-phase clinical investigations.

## Conclusions and perspectives

EMT is a crucial process in cancer metastasis, facilitating tumor progression, invasion, and drug resistance 371. Cells undergoing EMT frequently exhibit diminished sensitivity to chemotherapeutic agents, which contributes to treatment failure and disease relapse 260. Dysregulation of E3 ligases and DUBs contributes to the progression of EMT 372, which involves abnormal activation of EMT-TFs and the EMT-associated signaling pathways 50. Furthermore, accumulating evidence demonstrates that ubiquitination-mediated regulation of EMT significantly influences metastasis and chemoresistance in tumors. In this review, we comprehensively reviewed the mechanisms by which E3 ligases and DUBs regulate EMT and further emphasize the significance of ubiquitination-regulated EMT in tumor metastasis and chemoresistance. Furthermore, some preclinical and clinical evidence indicate that drugs targeting E3 ligases or DUBs have reversed EMT-induced metastasis and resistance in cancer.

Although drug development targeting ubiquitination factors holds significant promise for cancer therapy, the development of E3 or DUB inhibitors has encountered substantial challenges. The primary reason is due to the high structural and functional diversity of these enzymes, which complicates achieving inhibitor specificity and avoiding off-target effects that could lead to toxicity. Moreover, ubiquitination dynamics are influenced by cancer-specific mutations, microenvironmental factors, and heterogeneity in the EMT process, further exacerbating the complexity of inhibitor development. To address these challenges, future research should pursue innovative screening and development strategies, including: (1) Utilizing high-throughput assays and advanced cellular models to enable sensitive quantification of DUB activity and inhibition; (2) developing dual-action inhibitors targeting ubiquitination and EMT effectors simultaneously to counteract treatment resistance, building on recent progress in characterizing selective compounds against E3 ligases or DUBs; (3) validating predictive biomarkers in clinical cohorts for patient stratification and therapeutic optimization; (4) Employing emerging technologies, such as proximity-based labeling approaches for mapping ubiquitin dynamics, to resolve unanswered questions regarding ubiquitin signaling specificity; (5) identifying specific ubiquitination-EMT networks across diverse cancer types to clarify the mechanisms of ubiquitination-dependent EMT regulation in chemoresistance, as well as examining the universality and variability of this mechanism across different tumor types.

In conclusion, ubiquitination regulates tumor EMT through various mechanisms, thereby influencing tumor metastasis and treatment resistance. Targeting specific E3 ligases or DUBs reverses the EMT process, leading to sensitizing tumor cells to chemotherapeutic agents and suppressing distant metastasis. These findings provide novel insights into the mechanisms underlying tumor chemoresistance. Consequently, the development of E3 and DUB inhibitors offers a promising strategy for mitigating chemoresistance and metastasis in clinical oncology.

## Figures and Tables

**Table 1:** E3 ligases in EMT regulation

Protein	Substrates	Effect on EMT	Ref.
SPSB3	Snail	Negatively regulates EMT by degrading Snail	74
CHIP	Snail, Slug	Negatively regulates EMT by degrading Snail and Slug	75, 76
MARCH2	Snail	Negatively regulates EMT by promoting the degradation of Snail.	22
HECTD1	Snail	Negatively regulates EMT by degrading Snail.	92
Pellino-1	Snail, Slug	Positively regulates EMT by stabilizing Slug and Snail.	93, 94
MDM2	Snail, Slug, SMAD2/3	Positively regulates EMT by stabilizing Slug and Snail; activating the TGF-β signaling pathway.	77, 78, 163
A20 (TNFAIP3)	Snail	Positively regulates EMT by stabilizing Snail through monoubiquitination	96
β-TRCP1 (FBXW1)	Snail, Slug, Twist1, SMAD4, β-catenin	Negatively regulates EMT by degrading Slug, Snail, Twist1, and inhibiting TGF-β and Wnt/β-catenin signaling.	79, 80, 144, 158, 184
FBXW7 (FBW7)	Snail, ZEB1, ZEB2, Notch1	Negatively regulates EMT by regulating Snail, ZEB1, and ZEB2; inhibiting Notch signaling.	81, 131, 132
FBXL5	Snail	Negatively regulates EMT by degrading Snail.	82, 83
FBXL14 (Ppa)	Snail, Slug, Twist, ZEB2	Negatively regulates EMT by degrading Snail, Slug, ZEB2, and Twist.	84, 85, 145
FBXO3	Twist1	Positively regulates EMT by enhancing USP4-induced Twist1 stabilization.	148
FBXO11	Snail, ZEB1, β-catenin	Negatively regulates EMT by degrading Snail, ZEB1, and inhibiting Wnt/β-catenin signaling.	86, 128
FBXO28	Snail	Negatively regulates EMT by degrading Snail.	87
FBXO31	Snail	Negatively regulates EMT by degrading Snail.	88
FBXO45	Twist, Snail, Slug, and ZEB2	Negatively regulates EMT by degrading Twist, Snail, Slug, and ZEB2.	89
TRIM15	/	Positively regulates EMT by activating the Wnt/β-catenin signaling.	191
TRIM28	/	Positively regulates EMT by activating the Wnt/β-catenin signaling.	192
TRIM21	Snail	Negatively regulates EMT by degrading Snail.	90
TRIM26	ZEB1	Negatively regulates EMT by degrading ZEB1.	91, 129
TRIM46	Axin1	Positively regulates EMT by degrading Axin1 and activating the Wnt/β-catenin signaling	190
TRIM50	Snail	Negatively regulates EMT by degrading Snail.	91
TRIM59	RBPJ	Positively regulates EMT by stabilizing RBPJ and activating Notch signaling.	213
TRIM67	SMAD3	-Negatively regulates EMT by degrading SMAD3 and inhibiting TGF-β signaling. -Positively regulates EMT by activating Notch signaling.	159, 210
TRAF6	β-catenin	-Positively regulates EMT by degrading GSK3β and activating the Wnt/β-catenin signaling. -Negatively regulates EMT by inhibiting Wnt/β-catenin signaling.	188, 189
HERC3	SMAD7, EIF5A2	Positively regulates EMT by degrading Smad7 and activating TGF-β signaling Negatively regulates EMT by degrading EIF5A2.	161, 162
SIAH	ZEB1	Negatively regulates EMT by degrading ZEB1.	130
NEDD4L	TGF-β, TβRII, β-catenin, HIF1α	Negatively regulates EMT by inhibiting TGF-β, Wnt/β-catenin, and Hypoxia signaling.	160, 187
RBX1	Twist1	Positively regulates EMT by inhibiting FBXO45-induced Twist1 degradation.	147
RNF8	Slug, Twist1, GSK3β/β-catenin	Positively regulates EMT by stabilizing Slug and Twist1; activating Wnt/β-catenin signaling	95, 146, 193
RNF43	β-catenin	Negatively regulates EMT by inhibiting Wnt/β-catenin signaling.	183
RNF61 (MKRN1)	SNIP1	Positively regulates EMT by degrading SNIP1 and activating TGF-β signaling.	164
RNF111	SMAD3	Positively regulates EMT by activating TGF-β/SMAD3 signaling.	165, 166
TTC3	Smurf2	Positively regulates EMT by inhibiting Smurf2-induced TGFR and SMAD degradation and activating TGF-β signaling.	167
SPOP	Twist1	Negatively regulates EMT by degrading Twist1.	142
Smurf1	TGF-βRII	Negatively regulates EMT by degrading TGF-βRII and inhibiting TGF-β signaling.	154
Smurf2	SMAD1/2, TGF-βRI, HIF1α	Negatively regulates EMT by inhibiting TGF-β and Hypoxia signaling.	155-157, 227
Siah1-SIP-Skp1	β-catenin	Negatively regulates EMT by degrading β-catenin and inhibiting Wnt/β-catenin signaling.	186
VHL	HIF1α, HIF2α	Negatively regulates EMT by degrading HIF1α/ HIF2α and inhibiting Hypoxia signaling.	222, 223
Stub1	HIF2α	Negatively regulates EMT-associated vascular remodeling by degrading HIF2α and inhibiting Hypoxia signaling under acute hypoxia conditions.	229
DTX3	NICD	Negatively regulates EMT by degrading NICD and inhibiting Notch signaling.	206
WWP2	NICD	Negatively regulates EMT by downregulating Notch activity.	207
MIB1	/	Positively regulates EMT by activating Notch signaling.	211
BTRC	Twist1	Negatively regulates EMT by ubiquitination-mediated degradation of Twist1.	143

**Table 2:** DUBs in EMT regulation

Protein	Substrates	Effect on EMT	Ref.
USP1	Snail, TAK1	Positively regulates EMT by stabilizing Snail; activating TGF-β signaling by stabilizing TAK1.	97, 168
USP2	Snail	Positively regulates EMT by stabilizing Snail.	99
USP2a	TβRI/II	Positively regulates EMT by stabilizing TβRI/II and activating TGF-β signaling.	174
USP3	Snail, SUZ12	Positively regulates EMT by stabilizing Snail and SUZ12.	100, 175
USP4	Snail, Twist1, TβRI	Positively regulates EMT by stabilizing Snail and Twist1; activating TGF-β signaling.	101, 148, 169, 170
USP5	Snail, Slug, Twist1, β-catenin	Positively regulates EMT by stabilizing Snail, Slug, and Twist1; activating β-catenin signaling.	21, 122, 149, 196
USP7	β-catenin, HIF-1α	Positively regulates EMT by activating β-catenin and Hypoxia signaling.	197-199, 230
USP8	TβRII	Positively regulates EMT by stabilizing TβRII and activating TGF-β signaling.	176
USP9X	Snail, HIF-2α	Positively regulates EMT by stabilizing Snail; activating Hypoxia signaling.	102, 231
USP10	Snail, Slug, ZEB1, SMAD4	-Positively regulates EMT by stabilizing Snail and Slug; activating TGF-β signaling. -Negatively regulates EMT by degrading ZEB1.	103, 123, 140, 172
USP11	Snail, TβRII	Positively regulates EMT by stabilizing Snail; activating TGF-β signaling by stabilizing TβRII.	104, 177
USP13	Snail, Twist1, WISP1	Positively regulates EMT by stabilizing Snail and Twist1; activating Wnt/β-catenin signaling by stabilizing WISP1.	105, 150, 200
USP15	β-catenin	Positively regulates EMT by stabilizing β-catenin and activating Wnt/β-catenin signaling.	201
USP17	Snail, SMAD4	Positively regulates EMT by stabilizing Snail; activating TGF-β signaling.	106, 171
USP18	Snail, ZEB1, Twist1	Positively regulates EMT by stabilizing Snail, Twist1, and ZEB1.	107, 136, 151
USP19	TβRI	-Positively regulates EMT by USP19-CY, which stabilizes TβRI and TβRII to activate TGF-β signaling. -Negatively regulates EMT by USP19-ER, which inhibits EMT by reducing TβRI surface expression.	173
USP20	Slug	Positively regulates EMT by stabilizing Slug.	124
USP21	ZEB1	Positively regulates EMT by stabilizing ZEB1.	137
USP22	ZEB1	Positively regulates EMT by stabilizing ZEB1.	133, 195
	ADAM9	Negatively regulates EMT by stabilizing ADAM9 and Wnt/β-catenin signaling.	
USP25	β-catenin	Positively regulates EMT by stabilizing β-catenin and activating Wnt/β-catenin signaling.	202
USP26	Snail	-Positively regulates EMT by stabilizing Snail. -Negatively regulates EMT by stabilizing SMAD7 and inhibiting TGF-β signaling.	108, 178
USP27X	Snail	Positively regulates EMT by stabilizing Snail.	109
USP28	Snail, NICD	Positively regulates EMT by stabilizing Snail and NICD, activating Notch-induced EMT	110, 208
USP29	Snail, Twist1	Positively regulates EMT by stabilizing Snail and Twist1.	111, 152
USP30	Snail	Positively regulates EMT by stabilizing Snail.	112
USP34	Axin1	Negatively regulates EMT by stabilizing Axin1 and Wnt/β-catenin signaling.	194
USP35	Snail	Positively regulates EMT by stabilizing Snail.	113
USP36	DOCK4	Positively regulates EMT by stabilizing DOCK4 and activating Wnt/β-catenin signaling.	203
USP37	Snail, Gli-1	Positively regulates EMT by stabilizing Snail; activating Hh signaling.	114, 238
USP39	ZEB1	Positively regulates EMT by stabilizing ZEB1.	129
USP41	Snail	Positively regulates EMT by stabilizing Snail.	115
USP42	FZD	Negatively regulates EMT by stabilizing FZD and suppressing the Wnt signaling pathway.	182
USP43	ZEB1	Positively regulates EMT by stabilizing ZEB1.	138
USP47	Snail	Positively regulates EMT by stabilizing Snail.	116
USP51	ZEB1	Positively regulates EMT by stabilizing ZEB1.	134, 135
BRCC3	ZEB1	Positively regulates EMT by stabilizing ZEB1.	139
DUB3	Snail, Slug, Twist	Promotes EMT by stabilizing Snail, Slug, and Twist.	98, 125
OTUB1	Snail	Positively regulates EMT by stabilizing Snail.	117
OTUB2	β-catenin	Positively regulates EMT by stabilizing β-catenin and activating Wnt/β-catenin.	204
OTUD4	Snail	Positively regulates EMT by stabilizing Snail.	118
OTUD6B	VHL	Negatively regulates EMT by stabilizing VHL or mutated VHL, inhibiting Hypoxia signaling through downregulating HIF-1α or HIF-2α.	225, 226
Trabid	Twist1	Negatively regulates EMT by degrading Twist1	153
EIF3H	Snail	Positively regulates EMT by stabilizing Snail.	119
JOSD1	Snail	Positively regulates EMT by stabilizing Snail.	120
PSMD14	Snail	Positively regulates EMT by stabilizing Snail.	121

**Table 3:** E3 and DUBs promote metastasis by regulating EMT

Target	E3/DUBs		Regulatory mechanism	Ref.
Snail/Slug	Pellino-1	E3	Pellino-1 stabilizes Snail and Slug by K63-linked ubiquitin chains, facilitating lung tumorigenesis and metastasis *in vitro* and *in vivo*.	93, 94
Slug	RNF8	E3	RNF8 stabilizes Slug by K63-linked ubiquitin chains, promoting EMT and migration in LC cells* in vivo*.	95
Snail	A20	E3	A20 stabilizes Snail by monoubiquitination, thereby facilitating TGF-β1-induced EMT and metastasis in BC cells *in vivo*.	96
Snail	USP1	DUB	USP1 promotes the metastasis of OC cells by stabilizing Snail *in vitro* and *in vivo*.	97
Snail	USP2	DUB	USP2 promotes the proliferation and metastasis of choroidal melanoma cells by stabilizing the Snail protein.	99
Snail	USP4	DUB	USP4 stabilizes Snail via deubiquitination, driving EMT and metastasis in HCC *in vitro* and *in vivo*.	101
Snail/Slug	USP5	DUB	USP5 stabilizes Slug, promoting EMT and metastasis in bladder cancer and HCC *in vitro* and *in vivo*. USP5 stabilizes Snail, promoting EMT and metastasis in CRC cells *in vitro* and *in vivo*.	21, 122, 245
Snail	USP9X	DUB	USP9X stabilizes Snail, promoting the migration, invasion, and metastasis of TNBC cells *in vitro* and *in vivo.*	102
Snail/Slug	USP10	DUB	USP10 promotes EMT and the metastasis of BC cells by stabilizing Snail and Slug *in vitro* and *in vivo.*	103, 123
Snail	USP11	DUB	USP11 is significantly upregulated in OC tissues and promotes invasion and metastasis by deubiquitinating Snail *in vitro* and *in vivo*.	104
Snail	USP13	DUB	USP13 is highly expressed in GC and promotes EMT and metastasis by stabilizing Snail *in vitro* and *in vivo*.	105
Snail	USP17	DUB	USP17 promotes the migration and invasion of OSCC cells by stabilizing Snail *in vitro*.	106
Snail	USP18	DUB	USP18 is highly expressed in CRC tissues and promotes proliferation, migration and invasion by stabilizing Snail *in vitro*.	107
Slug	USP20	DUB	USP20 positively regulates Slug to promote BC metastasis and invasion *in vitro* and *in vivo.*	124
Snail	USP26	DUB	USP26 is highly expressed in ESCC and stabilizes Snail to promote the migration and invasion of ESCC cells *in vitro.*	108
Snail	USP27X	DUB	USP27X promotes the migration, invasion of BC cells by stabilizing Snail *in vitro.*	109
Snail	USP28	DUB	USP28 promotes BC metastasis by stabilizing Snail through deubiquitination *in vitro* and *in vivo.*	110
Snail	USP29	DUB	USP29 enhances the interaction between Snail and SCP1, stabilizing Snail and promoting the migration of GC cells *in vitro* and *in vivo.*	111
Snail	USP30	DUB	USP30 stabilizes Snail to facilitate the EMT and metastasis of BC cells *in vitro* and *in vivo.*	112
Snail	USP35	DUB	USP35 stabilizes Snail to facilitate the EMT and metastasis of GC tissues *in vitro* and *in vivo.*	113
Snail	USP37	DUB	USP37 stabilizes Snail via deubiquitination, promoting GC and LUAD cells metastasis *in vitro* and *in vivo*.	114, 249
Snail	USP41	DUB	USP41 increases the migration of breast cancer cells by stabilizing Snail, associated with poor prognosis in BC patients *in vitro* and *in vivo.*	115
Snail	USP47	DUB	USP47 stabilizes Snail to facilitate EMT and in CRC and BC cells *in vitro* and *in vivo.*	116, 246
Snail	DUB3	DUB	DUB3 stabilizes Snail to promote the EMT and metastasis of HCC and BC cells *in vitro* and *in vivo.*	248
Snail	OTUB1	DUB	OTUB1 is highly expressed in ESCC, which stabilizes Snail to promote ESCC metastasis *in vitro* and *in vivo.*	117
Snail	OTUD4	DUB	OTUD4 stabilizes Snail, which is identified as a novel therapeutic target for melanoma and BC metastasis *in vitro* and *in vivo.*	118, 247
Snail	JOSD1	DUB	JOSD1 is significantly overexpressed in LUAD, stabilizing Snail to promote EMT and metastasis *in vitro*.	120
Snail	PSMD14	DUB	PSMD14 stabilizes Snail to promote metastasis in ESCC *in vitro* and* in vivo*.	121
Snail	EIF3H	DUB	EIF3H interacts with and stabilizes Snail through deubiquitination, promoting EMT in ESCC *in vitro* and *in vivo.*	119
ZEB1	USP18	DUB	USP18 stabilizes ZEB1 through deubiquitination and enhances EMT and metastasis in ESCC *in vitro* and* in vivo*.	136
ZEB1	USP21	DUB	USP21 stabilizes ZEB1 to promote the EMT and metastasis of CRC *in vitro*, thereby contributing to poor prognosis.	137
ZEB1	USP22	DUB	USP22 stabilizes ZEB1 to enhance angiogenesis and EMT, thereby promoting the progression of OC *in vitro.*	133
ZEB1	USP39	DUB	USP39 stabilizes ZEB1 through deubiquitination, enhancing the proliferation and migration abilities of MM and HCC *in vitro* and* in vivo*.	129, 251.
ZEB1	USP43	DUB	USP43 is highly expressed in colorectal cancer tissues. It stabilizes ZEB1 to promote the migration and invasion of CRC *in vitro*.	138
ZEB1	USP51	DUB	USP51 plays an important role in the metastasis of GC, BC, and LUAD by stabilizing ZEB1 *in vitro* and* in vivo*.	134, 252
ZEB1	BRCC3	DUB	BRCC3 stabilizes ZEB1to promote EMT and metastasis in TNBC *in vitro* and* in vivo*.	139
Twist1	USP4	DUB	USP4 stabilizes Twist1 to drive EMT-associated stemness and malignancy in LC *in vitro* and TNBC invasion, migration, and tumor metastasis *in vitro* and* in vivo*.	148, 254
Twist1	USP13	DUB	USP13 stabilizes Twist1 to drive EMT, which promotes BC metastasis to the lung *in vitro* and* in vivo*.	150
Twist1	USP18	DUB	USP18 is highly expressed in GBM and promotes the migration and invasion of GBM cells by stabilizing Twist1 *in vitro* and* in vivo*.	151
Twist1	USP29	DUB	USP29 promotes the malignant phenotypes in TNBC cells by deubiquitinating Twist1 *in vitro* and* in vivo*.	152
Twist1	USP51	DUB	USP51 stabilizes Twist1 to drive EMT-associated stemness and malignancy in TNBC cells *in vitro*.	255
Twist1	RNF8	E3	RNF8 stabilizes Twist by K63-linked ubiquitin chains, promoting EMT, migration, and invasion in BC cells *in vitro* and* in vivo*.	146
Twist1	FBXO3	E3	FBXO3 stabilizes Twist1 by stabilizing USP4, enhancing BC cell migration and tumor metastasis *in vitro* and* in vivo*.	148
Twist1	RBX1	E3	RBX1 degrades FBXO45 to stabilize Twist1 and promotes the migration and invasion of TNBC both *in vitro* and *in vivo*.	147
**TGF-β signaling**				
SNIP1	RNF61 (MKRN1)	E3	RNF61 promotes TGF-β signaling by degrading SNIP1, facilitating EMT and metastasis in CRC *in vitro* and* in vivo*.	164
SMAD3	RNF111	E3	RNF111 activates TGF-β signaling by targeting SMAD3 to enhance Snail expression and promote NSCLC invasion and migration* in vitro.*	166
SMAD7	HERC3	E3	HERC3 induces SMAD7 degradation in an autolysosome-dependent manner, activating the TGF-β signaling and GBM cell invasion and tumor metastasis *in vitro* and *in vivo.*	161
SMAD2/3	MDM2	E3	MDM2 activates the Smad pathway to promote EMT during OC cell invasion and migration *in vitro.*	163
TAK1	USP1	DUB	USP1/WDR48 enhances TGF-β-mediated EMT and TNBC cell migration by stabilizing TAK1 *in vitro.*	168
TβRI	USP2a	DUB	USP2a activates TGF-β signaling by deubiquitinating TβRI, promoting metastasis in LC *in vivo.*	174
SUZ12	USP3	DUB	USP3 enhances TGF-β1-induced EMT and metastasis of GC cells by destabilizing SUZ12 *in vitro.*	175
TβRI	USP4	DUB	USP4 interacts with and deubiquitinates βRI, promoting TGF-β signaling-induced EMT and metastasis in HCC *in vitro* and *in vivo.*	170
TβRII	USP8	DUB	USP8 stabilizes TβRII to promote TGF-β/SMAD-induced EMT, invasion, and metastasis in BC cells *in vitro* and *in vivo.*	176
SMAD4	USP10	DUB	USP10 interacts with and stabilizes SMAD4 by removing Lys-48-linked ubiquitin chains, thereby promoting HCC metastasis *in vitro* and *in vivo.*	172
TβRII	USP11	DUB	USP11 stabilizes TβRII to activate the TGF-β signaling, promoting EMT and metastasis in BC *in vitro* and *in vivo.*	177
SMAD4	USP17	DUB	USP17 stabilizes SMAD4 to activate the TGF-β signaling, thereby promoting EMT and OS cell migration and invasion *in vitro.*	171
TβRI	USP19	DUB	USP19-CY (cytoplasmic isoform) stabilizes TβRI and TβRII to enhance TGF-β-induced EMT and migration in BC *in vitro*.	173
**Wnt/β-catenin signaling**				
GSK3β/ β-catenin	RNF8	E3	RNF8 inhibits GSK-3β and subsequently activates β-catenin signaling, promoting EMT and BC cell migration and tumor metastasis *in vitro* and *in vivo*	193
β-catenin	TRIM15	E3	TRIM15 activates the Wnt/β-catenin signaling to promote EMT, driving ESCC cell migration, invasion, and tumor metastasis *in vitro* and *in vivo.*	191
β-catenin	TRIM28	E3	TRIM28 activates the Wnt/β-catenin signaling to promote EMT, migration, and invasion of OC cells *in vitro.*	192
β-catenin	USP5	DUB	USP5 stabilizes β-catenin to activate the Wnt/β-catenin signaling, enhancing EMT and migration in NSCLC cells *in vitro.*	196
DDX3X, β-catenin	USP7	DUB	USP7 stabilizes β-catenin and DDX3X to activate Wnt/β-catenin signaling, promoting the metastasis of CRC and OS cells *in vitro.*	197, 198
WISP1	USP13	DUB	USP13 stabilizes WISP1 to activate the Wnt/β-catenin signaling, promoting ESCC cell migration and tumor metastasis *in vitro* and *in vivo*.	200
β-catenin	USP15	DUB	USP15 promotes the nuclear translocation of β-catenin and activates the Wnt/β-catenin signaling, enhancing the EMT and invasion in GC cells *in vitro.*	201
β-catenin	USP25	DUB	USP25 stabilizes β-catenin through interaction with TRIM21, activating β-catenin signaling-induced EMT and driving cell migration, invasion, and tumor metastasis *in vitro* and* in vivo.*	202
β-catenin	OTUB2	DUB	OTUB2 stabilizes β-catenin by suppressing TRAF6-mediated autophagy-dependent degradation, promoting Wnt/β-catenin signaling and driving EMT cell invasion and tumor metastasis *in vitro* and* in vivo.*	204

**Table 4:** E3 and DUBs promote resistance by regulating EMT

Drugs	Targeting	Drug resistance regulatory mechanism	Cancers	Ref
Cisplatin	NEDD4L	Cisplatin induces a decrease in NEDD4, stabilizing CPT1CAK to drive EMT and cisplatin resistance *in vitro.*	NSCLC	263
	FBXW7	Lower expression of FBXW7 stabilizes Snail, driving EMT and cisplatin resistance *in vitro.*	NSCLC	265
	PARK2	Cisplatin inhibits PARK2-mediated vimentin, promoting EMT and cisplatin resistance *in vitro* and* in vivo.*	LUAD	266
	TRAF6	TRAF6 degrades GSK3β, thereby activating β-catenin signaling and promoting EMT and cisplatin resistance *in vitro* and* in vivo.*	NPC	188
	Hakai	Stabilizes p-AKT to enhance EMT and cisplatin resistance *in vitro.*	NSCLC	267
	USP1	USP1 stabilizes Snail to enhance EMT and cisplatin resistance in* vitro.*	OC	97
	USP7	USP7 stabilizes c-Myc to enhance EMT and cisplatin resistance *in vitro*.	LUAD	271
	USP9X	USP9X stabilizes Snail to enhance EMT and cisplatin resistance *in vitro* and* in vivo.*	TNBC	102
	USP22	USP22 stabilizes c-Myc and ALDH1A3 to enhance EMT and cisplatin resistance *in vitro* and* in vivo.*	TNBC, LUAD	272, 273
	USP27X	USP27X stabilizes Snail to enhance EMT and cisplatin resistance *in vitro* and* in vivo.*	TNBC	109
	USP29	USP29 stabilizes Twist1 to enhance EMT and cisplatin resistance *in vitro* and* in vivo.*	TNBC	152
	USP32	USP32 stabilizes SMAD2 to enhance EMT and cisplatin resistance *in vitro* and* in vivo.*	GC	270
	USP37	USP37 stabilizes Gli-1 to enhance EMT and cisplatin resistance *in vitro* and* in vivo.*	BC	238
	USP51	USP51 stabilizes ZEB1 to enhance EMT and cisplatin resistance *in vitro.*	LC	269
	PSMD14	PSMD14 stabilizes Snail to enhance EMT and cisplatin resistance *in vitro* and* in vivo.*	ESCC	121, 268
Oxaliplatin	OTUB2	OTUB2 stabilizes SP1 and GINS1 to enhance EMT and Oxaliplatin resistance *in vitro* and* in vivo.*	CRC	275
	FBXW7	Lower expression of FBXW7 stabilizes ZEB2, driving EMT and Oxaliplatin resistance *in vitro* and *in vivo.*	CRC	132
Doxorubicin	RNF8	RNF8 stabilizes Twist via K63-linked polyubiquitin chains, driving EMT and Dox resistance *in vivo*.	TNBC	[146
	SIAH1	Dox induces SIAH1 decrease, stabilizing ZEB1 to drive EMT and Dox resistance *in vitro.*	OS, HCC	278, 279
	USP9X	USP9X stabilizes Snail to enhance EMT and Dox resistance.	TNBC	109
	USP14	USP14 modulates Wnt signaling to enhance Dox resistance *in vitro*.	MM	282
	USP29	USP29 stabilizes Snail to enhance EMT and Dox resistance *in vitro* and *in vivo.*	NSCLC	283
	USP45	USP29 stabilizes MYC to enhance EMT and Dox resistance *in vivo.*	CC	284
Gemcitabine	UBR5	UBR5 degrades O-GlcNAcase (OGA), inducing EMT and GEM resistance *in vitro* and *in vivo.*	PC	286
	TRIM59	TRIM59 stabilizes RBPJ, activating Notch signaling to induce EMT and drive GEM *in vitro* and *in vivo.*	PC	213
	RNF126	RNF126 ubiquitinates and degrades PTEN, activating the AKT/GSK-3β/β-catenin pathway to induce EMT and drive GEM resistance *in vitro* and *in vivo.*	PC	287
	FBW7	MiR-223 downregulates FBW7 to activate Notch-1-induced EMT and GEM resistance *in vitro.*	PC	209
	Smurf2	miR-15b downregulates Smurf2 to stabilize Smad2/3, activating the TGF-β-induced EMT and driving resistance *in vitro*.	PC	289
Paclitaxel	USP29	USP29 stabilizes Snail to enhance EMT and paclitaxel resistance *in vitro*	NSCLC	283
	USP30	USP30 stabilizes Snail to enhance EMT and paclitaxel resistance *in vitro* and *in vivo.*	BC	112
5-Fu	FBXW7	Lower expression of FBXW7 stabilizes ZEB2, driving EMT and Oxaliplatin resistance *in vitro* and *in vivo.*	CRC	132
Sunitinib	TRIM21	TRIM21 stabilizes AXL to enhance sunitinib resistance *in vitro* and *in vivo.*	KIRC	295
Sorafenib	RNF8	RNF8 upregulates N-cadherin and Snail to enhance sorafenib resistance *in vitro.*	HCC	294
Lenvatinib	RNF8	RNF8 upregulates N-cadherin and Snail to enhance lenvatinib resistance *in vitro.*	HCC	294
Temozolomide	HERC3	HERC3 degrades SMAD7 to activate TGF-β signaling, inducing EMT and autophagy, and TMZ resistance *in vitro* and *in vivo*	GBM	161
Vemurafenib	OTUD4	OTUD4 stabilizes Snail to enhance EMT and Vemurafenib resistance *in vitro*.	MEL	118
PLX-4720	OTUD4	OTUD4 stabilizes Snail to enhance EMT and PLX-4720 resistance *in vitro.*	MEL	118

**Table 5:** Summary of pharmacological strategies directly targeting the E3 ligase or DUBs for cancer therapy in clinical trials

Target	Drug	Mechanism	Cancer types	Phase	Identifier
**Drug targeting E3 ligases**					
CRBN	ARV-110	An inhibitor of PROTACs with E3 CRBN, targeting the androgen receptor.	Prostate cancer	Phase I Phase II	NCT05177042, NCT03888612
	ARV-471	An inhibitor of PROTACs with E3 CRBN, targeting the estrogen receptor.	Breast cancer	Phase I Phase II Phase III	NCT04072952, NCT05548127, NCT05732428, NCT05573555, NCT05654623, NCT05501769, NCT06125522, NCT05463952
	CC92480 (Mezigdomide)	A novel E3 ligase CRBN modulator, targeting IKZF1 and ZFP91.	Multiple myeloma	Phase I Phase II	NCT05707390, NCT06645678, NCT07032714, NCT05552976, NCT06163898, NCT06988488, NCT05519085, NCT03989414, NCT03374085
VHL	HP518	Recruits VHL to ubiquitinate and degrade the AR protein.	Prostate cancer	Phase I Phase II	NCT05252364, NCT06155084
CRL4-CRBN	CC-90009	Recruits the CRL4-CRBN E3 complex to ubiquitinate and degrade GSPT1.	Acute myeloid leukemia	Phase I Phase II	NCT04336982, NCT02848001
	KPG-818	Specifically binds to CRBN, modulating the activity of the CRL4-CRBN complex.	Hematological malignancies	Phase I	NCT04283097
Cbl-b	NX-1607	Binds to Cbl-b, preventing activation and inhibiting function.	Advanced malignancies	Phase 1	NCT05107674
MDM2	RG7112	Binds to MDM2 and disrupts MDM2-p53 interaction, stabilizing p53.	Myelogenous leukemia, Neoplasms, Sarcoma Hematologic neoplasms,	Phase I	NCT01677780, NCT00559533, NCT00623870, NCT01143740, NCT01164033, NCT01605526
	APG115	Disrupts MDM2-p53 interaction, inducing cell-cycle arrest and apoptosis.	Solid tumor or lymphoma	Phase I	NCT02935907, CTR20170975
	JNJ-26854165 (Serdemetan)	Inhibits HDM2-P53 interaction, stabilizing p53.	Neoplasms	Phase I	NCT00676910
	ALRN-6924	Binds to MDM2 and MDMX to disrupt MDM2-p53 interaction, stabilizing p53.	Leukemia, Solid Tumors	Phase I	NCT03654716, NCT02909972, NCT02264613, NCT03725436, NCT05622058
	RG7388 (Idasanutlin)	Binds to MDM2 to disrupt MDM2-p53 interaction, stabilizing p53.	Acute myeloid leukemia, Solid tumors	Phase I	NCT02670044, NCT03362723, NCT02828930, NCT01773408, NCT01462175, NCT01901172
	MK-8242	Prevents HDM2-P53 interaction, stabilizing p53.	Solid tumors, Acute myeloid leukemia	Phase I	NCT01463696, NCT01451437
	SAR405838	High-affinity binds to MDM2 to disrupt MDM2-p53 interaction, stabilizing p53.	Neoplasm malignant	Phase I	NCT01636479, NCT01985191
	CGM097	Binds to MDM2 to disrupt MDM2-p53 interaction, stabilizing p53.	Solid tumors	Phase I	NCT01760525
	Milademetan (DS-3032b)	Specifically binds to MDM2 to disrupt MDM2-p53 interaction, stabilizing p53.	Advanced solid tumor, Myeloid leukemia, Myeloma, Dedifferentiated liposarcoma	Phase I	NCT01877382, NCT03671564, NCT03614455, NCT02579824, NCT04979442
	Siremadlin (HDM-201)	Binds to the binding pocket of p53 in MDM2, competitively inhibiting MDM2-p53 interaction to stabilize p53.	Colorectal cancer, Solid and hematological tumors, Liposarcoma	Phase I	NCT02890069, NCT02143635, NCT02343172
	AMG232	Binds to MDM2 to disrupt MDM2-p53 interaction, stabilizing p53.	Malignancy	Phase I	NCT01723020, NCT02110355, NCT02016729
	RO6839921	An inactive pegylated prodrug of the oral MDM2 antagonist to disrupt MDM2-p53 interaction, stabilizing p53.	Acute myeloid leukemia	Phase I	NCT02098967
	BI907828	Binds to MDM2 to disrupt MDM2-p53 interaction, stabilizing p53.	Different types of advanced cancer, Glioblastoma	Phase I	NCT03449381, NCT05376800, NCT03964233
IAPs	GDC-0152	Binds to BIR domains of ML-IAP, XIAP, cIAP1, and cIAP2 to degrade cIAP1, promoting cell apoptosis.	Advanced or metastatic malignancies	Phase I	NCT00977067
	LCL161	Binds to the BIR3 domain of cIAPs, inducing their autoubiquitination and degradation.	Colorectal cancer, Multiple myeloma, Solid tumors, Neoplasms, Small cell lung cancer, Breast cancer	Phase I Phase II	NCT02890069, NCT03111992, NCT01240655, NCT01968915, NCT01617668, NCT01098838, NCT02098161, NCT02649673
	AT-406	Binds to XIAP and cIAPs, inducing cIAP1 degradation and caspase activation.	Adenocarcinoma of the pancreas, Squamous cell carcinoma, Solid tumors	Phase I Phase II	NCT04122625, NCT03871959, NCT02022098, NCT03270176, NCT01078649
	TL-32711	Binds to the BIR3 domain of cIAPs, inducing their autoubiquitination and degradation	Ovarian cancer	Phase I	NCT01940172
	APG-1387	A next-generation IAP inhibitor mimics endogenous SMAC to degrade IAPs.	Solid tumors	Phase I	NCT03386526
	AEG-40826	A selectively inhibits IAP biological activity, restores apoptotic signaling.	Advanced solid tumors	Phase I	NCT00708006
	BI-891065	Binds to CIAPs and promotes their degradation, inducing tumor cell apoptosis.	Neoplasm, Non-small-cell lung carcinoma	Phase I	NCT04138823, NCT03166631
CRLs	MLN4924 (Pevonedistat)	Blocks the activation of NEDD8 by competitively binding to the adenosineylation site of NAE, thereby inhibiting cullin ring neddylation.	Multiple myeloma, Myeloid leukemia, Mesothelioma, Solid neoplasm, Lymphoblastic leukemia, Metastatic melanoma	Phase I Phase II	NCT03770260, NCT04712942, NCT03319537, NCT03330106, NCT03814005, NCT03349281, NCT02610777, NCT02782468, NCT03486314, NCT03459859, NCT01862328, NCT01814826, NCT03057366, NCT02122770, NCT00911066, NCT00722488, NCT00677170, NCT01011530
**Drug targeting DUBs**					
USP14 UCHL5	VLX1570	Binds to USP14 and UCHL5 to inhibit their function.	Multiple myeloma	Phase I Phase II	NCT02372240
USP1	KSQ-4279	A selective small molecule inhibitor of USP1 with anti-proliferative activity in tumors with HRR mutations.	Advanced solid tumors	Phase I	NCT05240898
	TNG348	A reversible allosteric inhibitor, inhibiting its deubiquitinase activity by binding to the allosteric site of USP1	BRCA1/2 mutant tumors or HRD+ solid tumors	Phase I Phase II	NCT06065059
	XL309	A selective USP1 inhibitor with an unknown binding mechanism	Advanced solid tumors	Phase I	NCT05932862
	SIM0501	A selective USP1 inhibitor with an unknown binding mechanism	Advanced solid tumors	Phase I	NCT06331559
	HSK39775	A selective USP1 inhibitor with an unknown binding mechanism	Advanced solid tumors	Phase I Phase II	NCT06314373
UCHL3	Perifosine	A selective UCHL3 inhibitor with an unknown binding mechanism	Pediatric solid tumors, Refractory tumors, Leukemia, Breast cancer, Colorectal cancer	Phase I Phase II Phase III	NCT01049841, NCT00873457, NCT00391560, NCT00054145, NCT01097018

## Abbreviations

5-FU: 5-Fluorouracil
ACF7: actin crosslinking factor 7
AML: acute myeloid leukemia
ATXN1: ataxin-1
AXL: AXL receptor tyrosine kinase
AR: androgen receptor
AXIN: axis inhibitory protein
BC: breast cancer
BCR: B cell receptor
BIRC5: baculoviral IAP repeat containing 5
BRCA1: breast cancer susceptibility gene 1
CAFs: cancer-associated fibroblasts
ccRCC: clear cell renal cell carcinoma
CDK4/6: cyclin-dependent kinase 4/6
CHIP: C-terminus of HSC70-interacting protein
CIC: cancer-initiating cell
CLL: chronic lymphocytic leukemia
CPT1C: carnitine palmitoyltransferase 1C
CRC: colorectal cancer
CRLs: cullin-RING ligases
CSC: cancer stem cell
CXCR4: C-X-C motif chemokine receptor 4
CRL4-CRBN: cullin4-RING E3 ubiquitin ligase-cereblon complex
DUB: deubiquitinating enzyme
Dox: doxorubicin
EBV: epstein-barr virus
EMT: epithelial-mesenchymal transition
ER: estrogen receptor
ESCC: esophageal squamous cell carcinoma
FAT10: HLA-F adjacent transcript 10
FBX: F-box protein
FBXW: F-box and WD repeat domain containing
FBXL: F-box and leucine-rich repeat protein
FZD: frizzled
GBM: glioblastoma
GC: gastric cancer
Gli: glioma-associated oncogene homolog
GSK-3β: glycogen synthase kinase-3β
GSPT1: G1-to-S phase transition 1
HCC: hepatocellular carcinoma
HECT: homologous to E6AP carboxyl terminus
HECTD1: HECT domain E3 ubiquitin protein ligase 1
HERC3: HECT and RLD domain containing E3 ubiquitin protein ligase 3
Hh: hedgehog
HIF: hypoxia-inducible Factor
HRD+: homologous recombination deficiency positive
IAP: inhibitor of apoptosis protein
iCCA: intrahepatic cholangiocarcinoma
KIRC: kidney renal clear cell carcinoma
LC: lung cancer
LOX: lysyl oxidase
LUAD: lung adenocarcinoma
MARCH: membrane-associated RING-CH protein
MDM2: murine double minute 2
MGD: molecular glue degrader
MIB1: MIB E3 ubiquitin protein ligase 1
MM: multiple myeloma
MMPs: matrix metalloproteinases
mCRPC: metastatic castration-resistant prostate cancer
NAE: NEDD8-activating enzyme
NEDD4L: neural precursor cell expressed developmentally downregulated 4-Like
NEDD8: neural precursor cell expressed developmentally downregulated 8
NICD: notch intracellular domain
NSCLC: non-small cell lung cancer
OC: ovarian cancer
OGA: O-GlcNAcase
OS: osteosarcoma
OSCC: oral squamous cell carcinoma
OTU: ovarian tumor-related protease
OTUD6B: ovarian tumor domain-containing 6B
PARK2: parkin RBR E3 ubiquitin protein ligase
PC: pancreatic cancer
PCa: prostate cancer
PD-L1: programmed cell death ligand 1
PELI1: pellino1
PROTAC: proteolysis-targeting chimera
PSMD14: proteasome non-ATPase regulatory subunit 14
PTEN: phosphatase and tensin homolog
PTM: post-translational modification
RBR: RING-Between-RING
RBPJ: recombination signal binding protein for immunoglobulin Kappa J region
RCC: renal cell carcinoma
RING: really interesting new gene
RNF: ring finger protein
SCLC: small cell lung cancer
SMAC: second mitochondrial-Derived activator of caspases
SIAH1: seven in absentia homolog 1
SIP: siah-interacting protein
SMAD: small mother against decapentaplegic
Smo: smoothened
Snail: snail family transcriptional repressor
SPOP: speckle-type POZ protein
STAT3: signal transducer and activator of transcription 3
STAMBPL1: STAM binding protein like 1
STUB1: STIP1 homology and U-box containing protein 1
SUZ12: suppressor of zeste 12
TAK1: transforming growth factor β-activated kinase 1
TβRI: TGF-β type I receptor
TβRII: TGF-β type II receptor
TGF-β: transforming growth factor β
TMZ: temozolomide
TNBC: triple-negative breast cancer
TRIM: tripartite motif
TRAF6: TNF receptor associated factor 6
Twist: twist family BHLH transcription factor
Ub: Ubiquitin
UBR5: ubiquitin ligase E3 component N-recognition protein 5
UCHL5: ubiquitin carboxyl-terminal hydrolase isozyme L5
UPS: ubiquitin-proteasome system
USP: ubiquitin-specific protease
VHL: von hippel-lindau
WM: waldenström macroglobulinemia
WWP2: WW domain containing E3 ubiquitin protein ligase 2
XIAP: X-linked inhibitor of apoptosis protein
ZEB1/2: zinc finger E-box binding homeobox 1/2
β-TrCP: β-transducin repeat-containing protein
UCHs: ubiquitin carboxy-terminal hydrolase
MINDYs: motif interacting with ubiquitin-containing novel DUB
JAMMs: JAMM/MPN domain-associated metallopeptidases
MJDs: machado-joseph domain proteases
ZUFSP: Zinc finger and UFSP domain protein
SPSB3: SPRY Domain-Containing SOCS Box Protein 3
MARCH2: membrane associated ring-CH-type finger 2
